# Supplementary figures and images for: Tau phosphorylation suppresses oxidative stress-induced mitophagy via FKBP8 receptor modulation
Source: PLoS One. 2025 Jan 3;20(1):e0307358. doi: 10.1371/journal.pone.0307358 (PMC11698316; doi:10.1371/journal.pone.0307358)

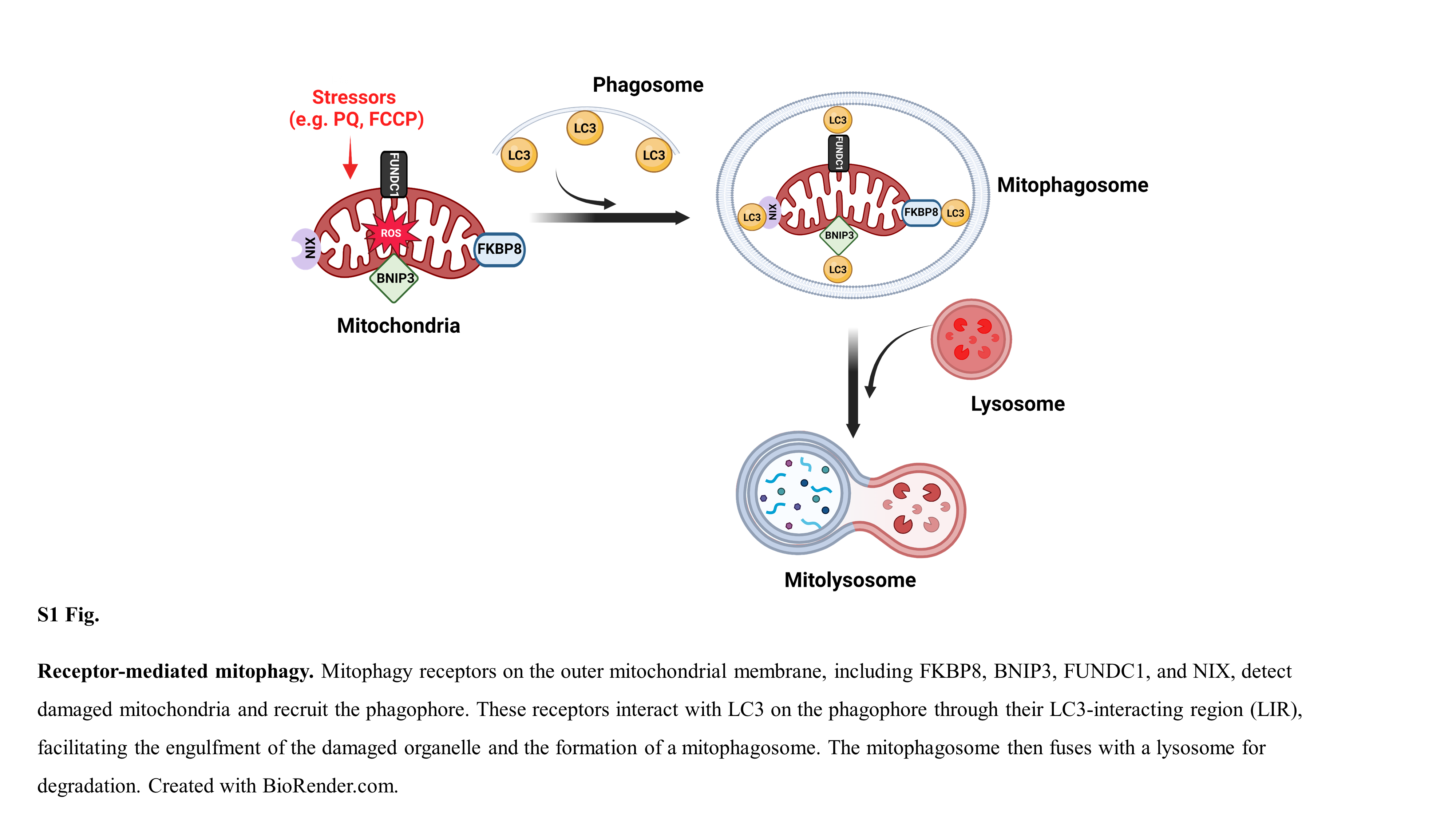

Supplement: S1 Fig — Mitophagy receptors on the outer mitochondrial membrane, including FKBP8, BNIP3, FUNDC1, and NIX, detect damaged mitochondria and recruit the phagophore. These receptors interact with LC3 on the phagophore through their LC3-interacting region (LIR), facilitating the engulfment of the damaged organelle and the formation of a mitophagosome. The mitophagosome then fuses with a lysosome for degradation. Created with BioRender.com. (TIF) [file pone.0307358.s001.TIF]

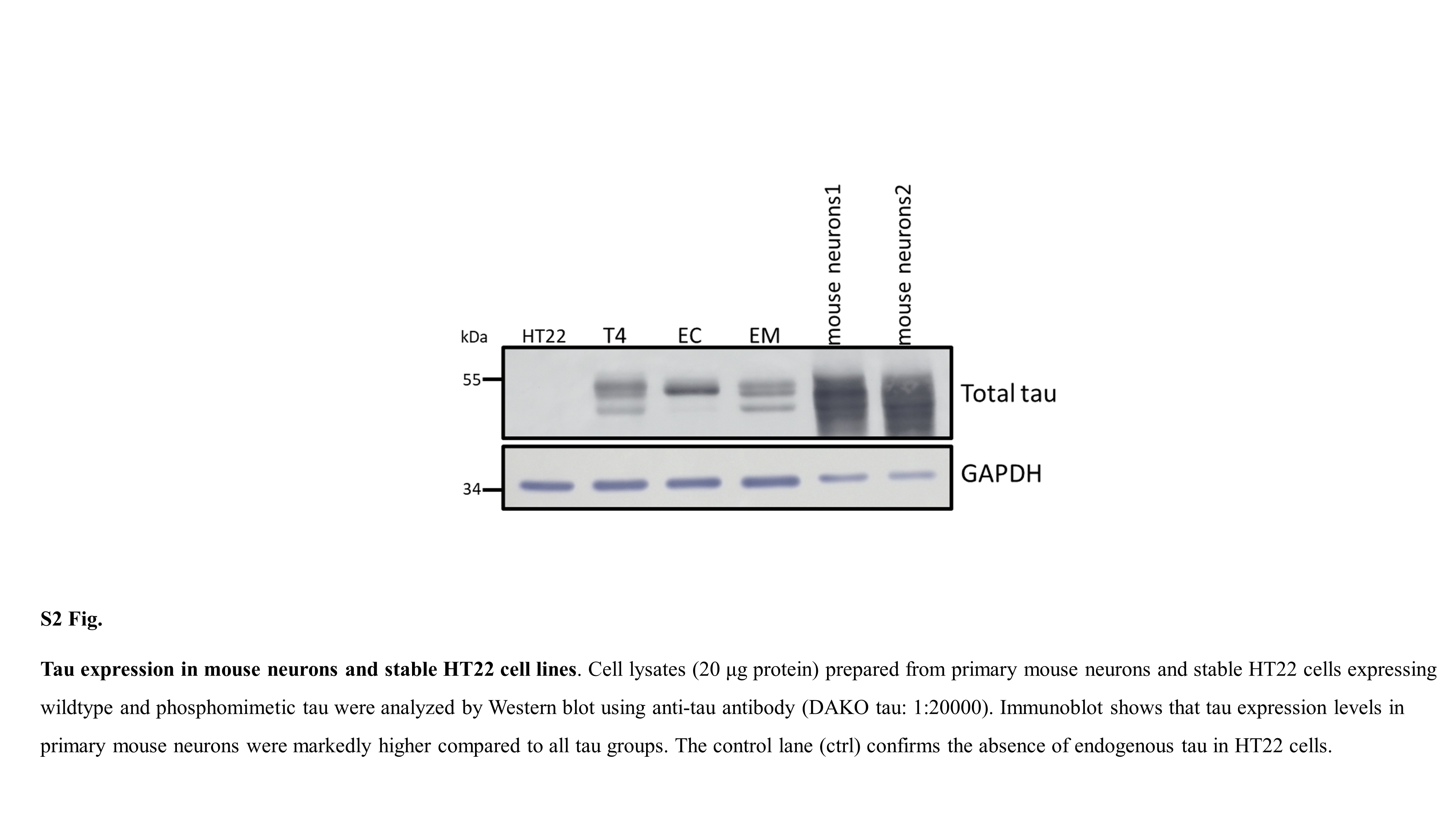

Supplement: S2 Fig — Cell lysates (20 μg protein) prepared from primary mouse neurons and stable HT22 cells expressing wildtype and phosphomimetic tau were analyzed by Western blot using anti-tau antibody (DAKO tau: 1:20000). Immunoblot shows that tau expression levels in primary mouse neurons were markedly higher compared to all tau groups. The control lane (ctrl) confirms the absence of endogenous tau in HT22 cells. (TIF) [file pone.0307358.s002.TIF]

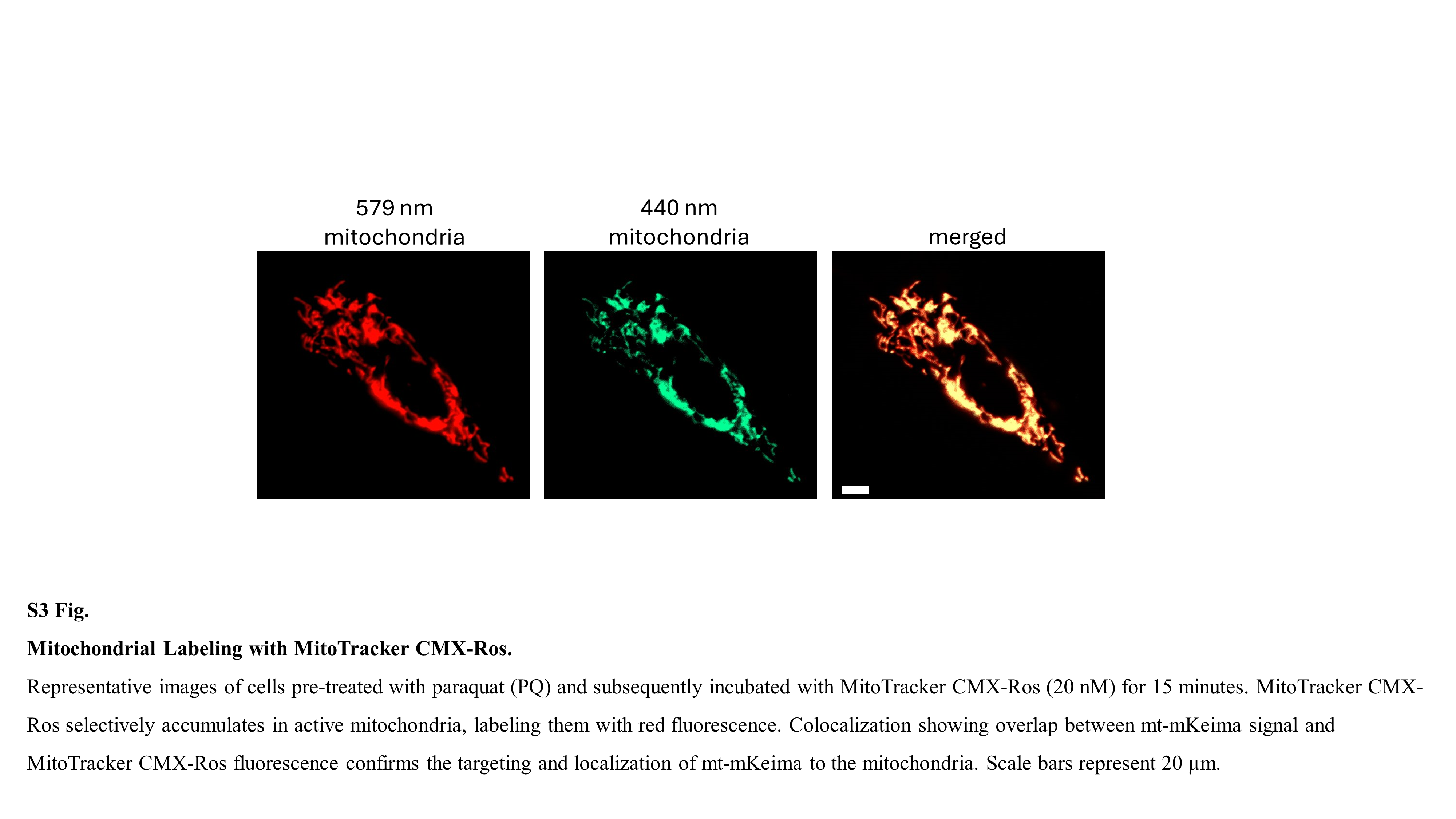

Supplement: S3 Fig — Representative images of cells pre-treated with paraquat (PQ) and subsequently incubated with MitoTracker CMX-Ros (20 nM) for 15 minutes. MitoTracker CMX-Ros selectively accumulates in active mitochondria, labeling them with red fluorescence. Colocalization showing overlap between mt-mKeima signal and MitoTracker CMX-Ros fluorescence confirms the targeting and localization of mt-mKeima to the mitochondria. Scale bars represent 20 μm. (TIF) [file pone.0307358.s003.TIF]

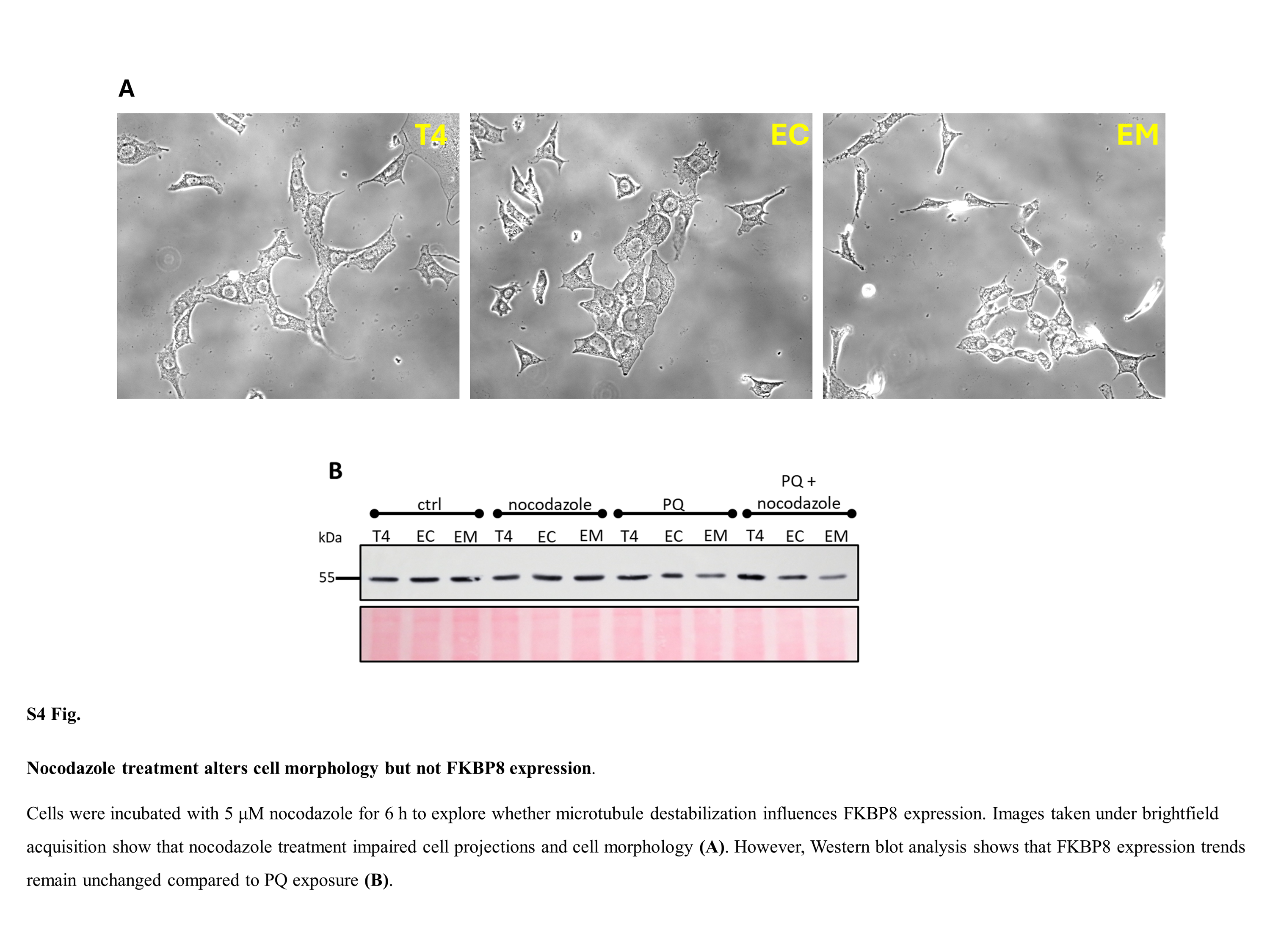

Supplement: S4 Fig — Cells were incubated with 5 μM nocodazole for 6 h to explore whether microtubule destabilization influences FKBP8 expression. Images taken under brightfield acquisition show that nocodazole treatment impaired cell projections and cell morphology (A). However, Western blot analysis shows that FKBP8 expression trends remain unchanged compared to PQ exposure (B). (TIF) [file pone.0307358.s004.tif]

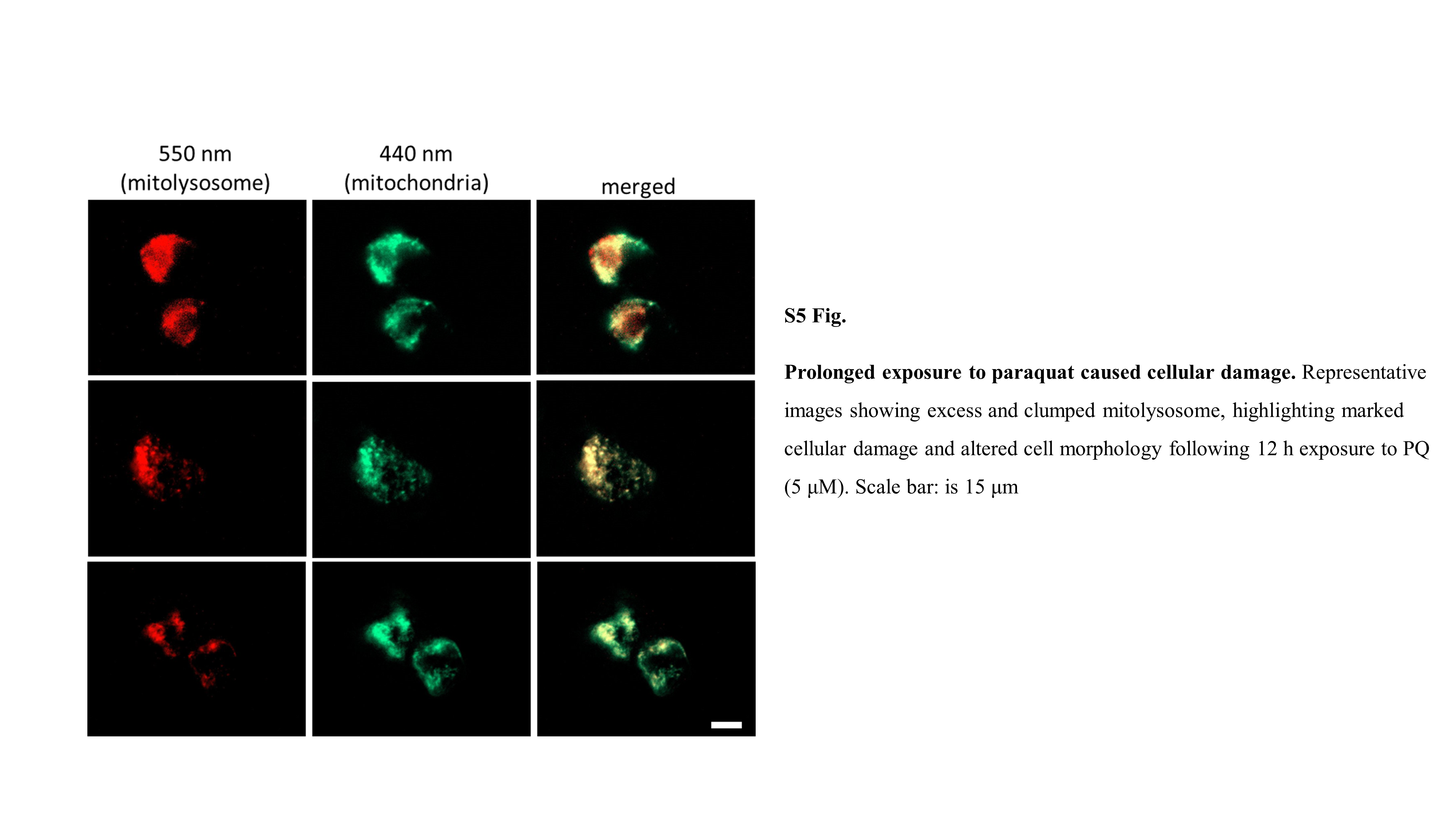

Supplement: S5 Fig — Representative images showing excess and clumped mitolysosome, highlighting marked cellular damage and altered cell morphology following 12 h exposure to PQ (5 μM). Scale bar: is 15 μm. (TIF) [file pone.0307358.s005.TIF]

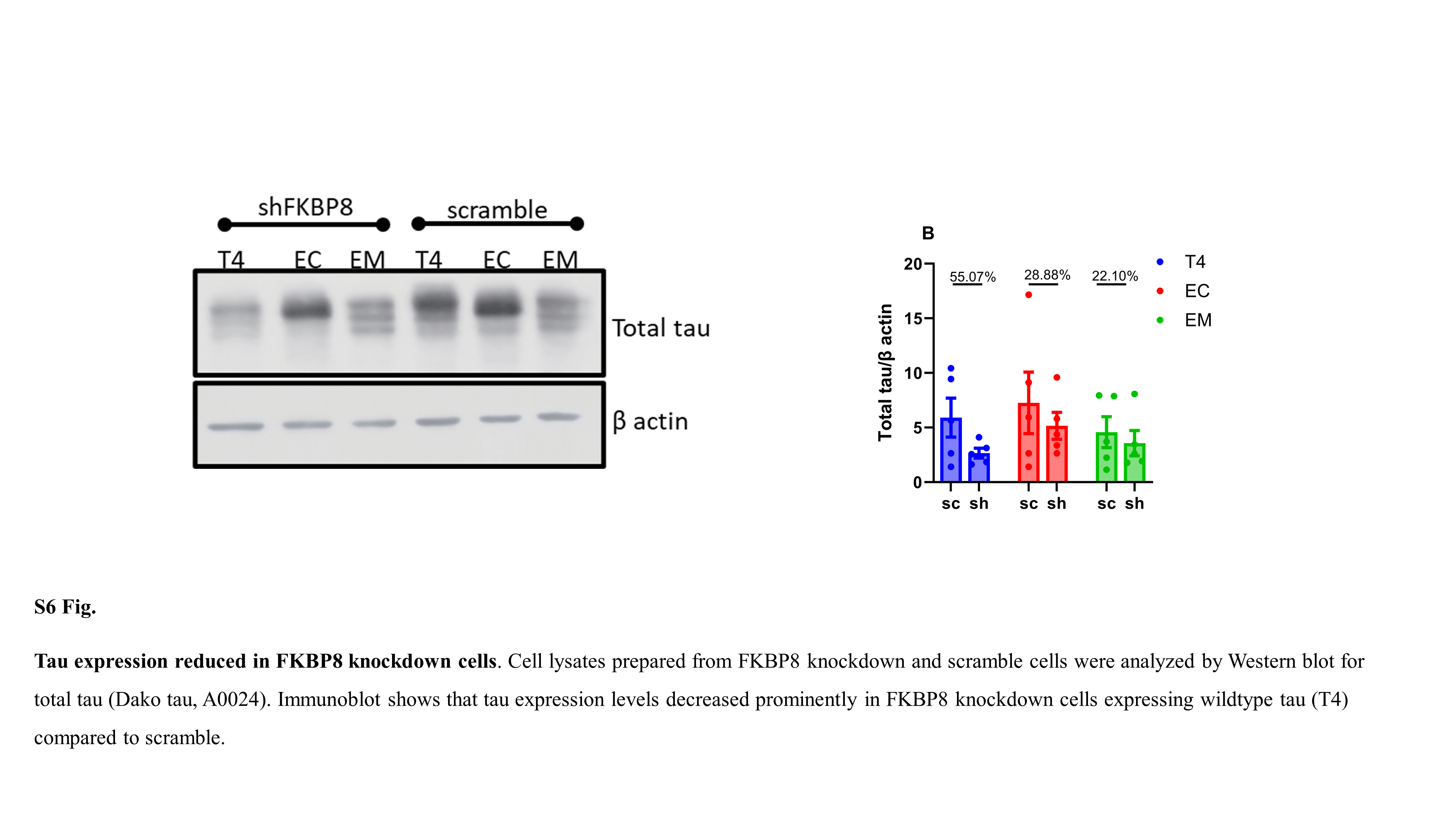

Supplement: S6 Fig — Cell lysates prepared from FKBP8 knockdown and scramble cells were analyzed by Western blot for total tau (Dako tau, A0024). Immunoblot shows that tau expression levels decreased prominently in FKBP8 knockdown cells expressing wildtype tau (T4) compared to scramble. (TIF) [file pone.0307358.s006.TIF]
