# Supplementary material for: Tau phosphorylation suppresses oxidative stress-induced mitophagy via FKBP8 receptor modulation
Source: PLoS One. 2025 Jan 3;20(1):e0307358. doi: 10.1371/journal.pone.0307358 (PMC11698316; doi:10.1371/journal.pone.0307358)
Supplement: S1 Raw images — (PDF) [file pone.0307358.s007.pdf]

Uncropped blots

All signals were detected with Immobilon Crescendo Western HRP-substrate (Millipore, WBLUR0500) and images captured with a Kwik Quant imager (Kindle Biosciences, LLC)

Figure 1. Total Tau

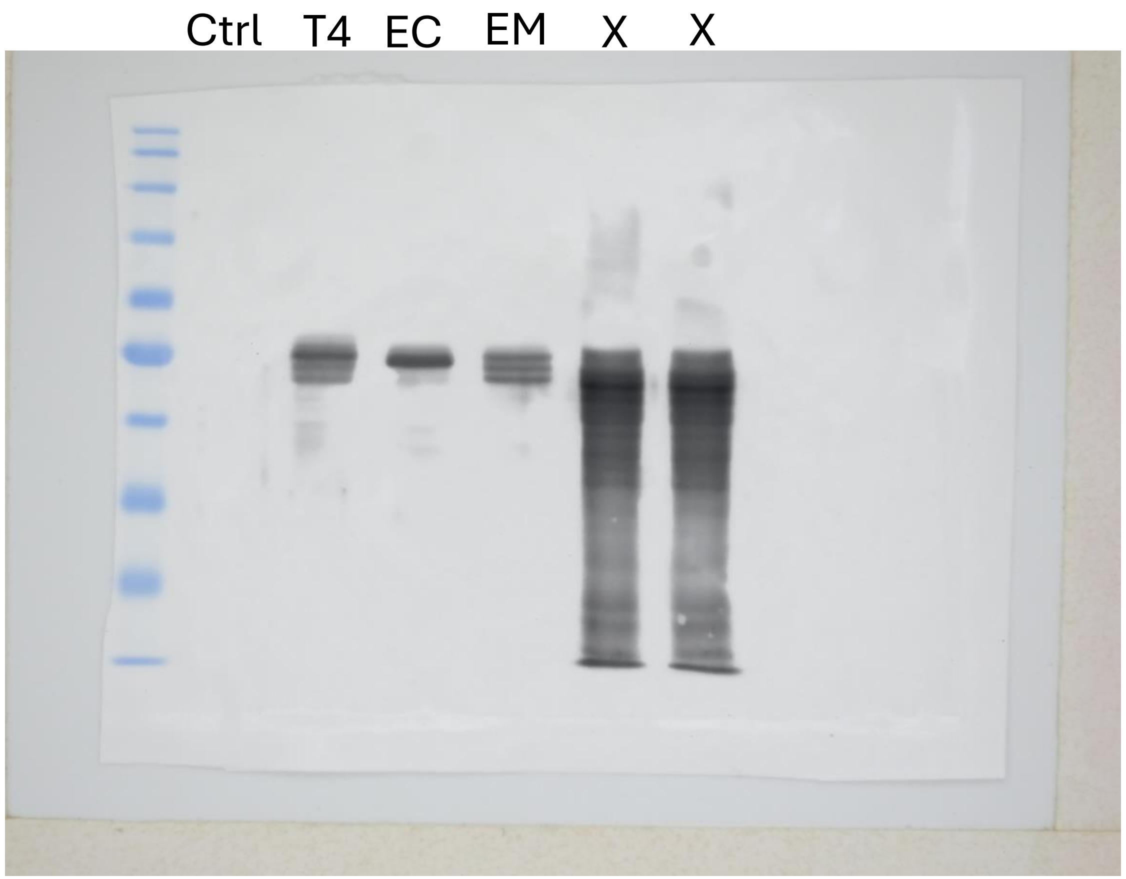

Figure 1. pT231/p235

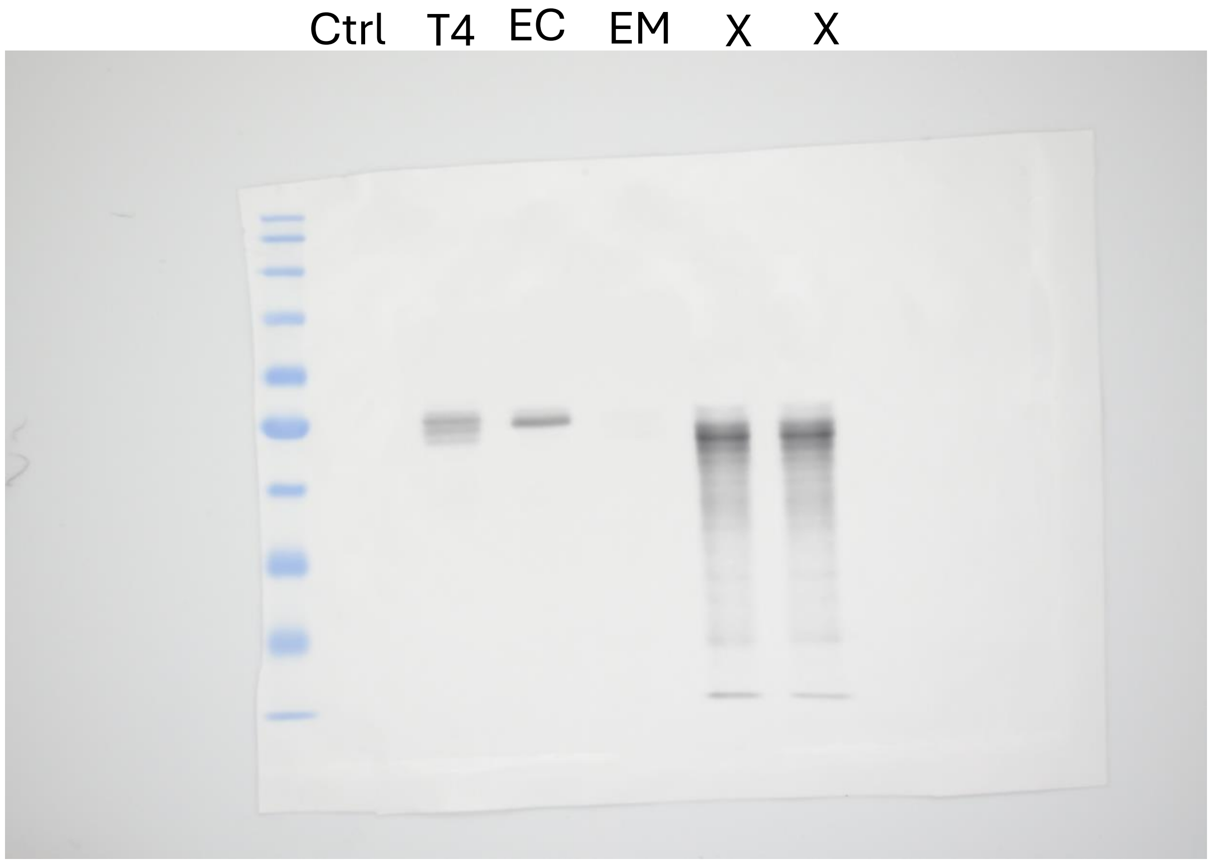

Figure 1. pS396/404

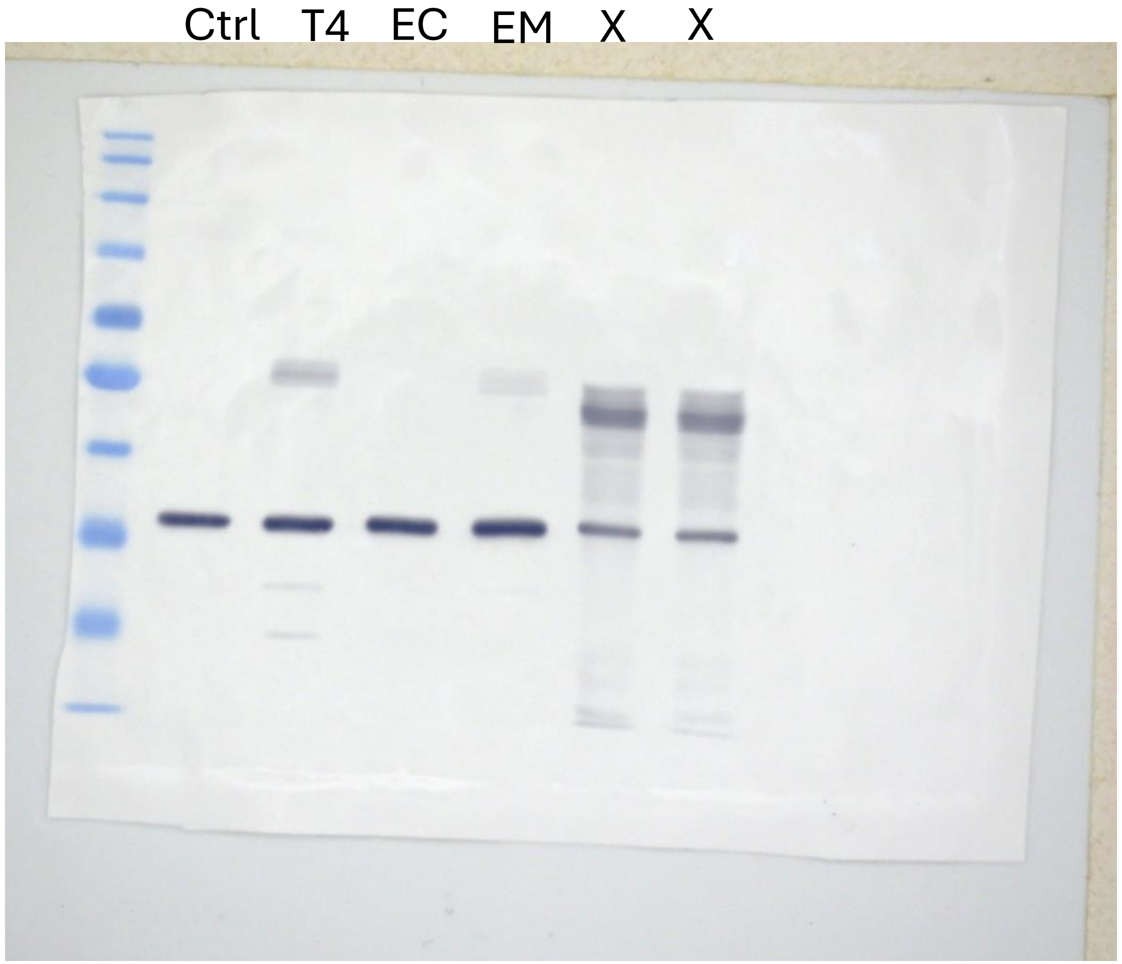

Figure 1. GAPDH

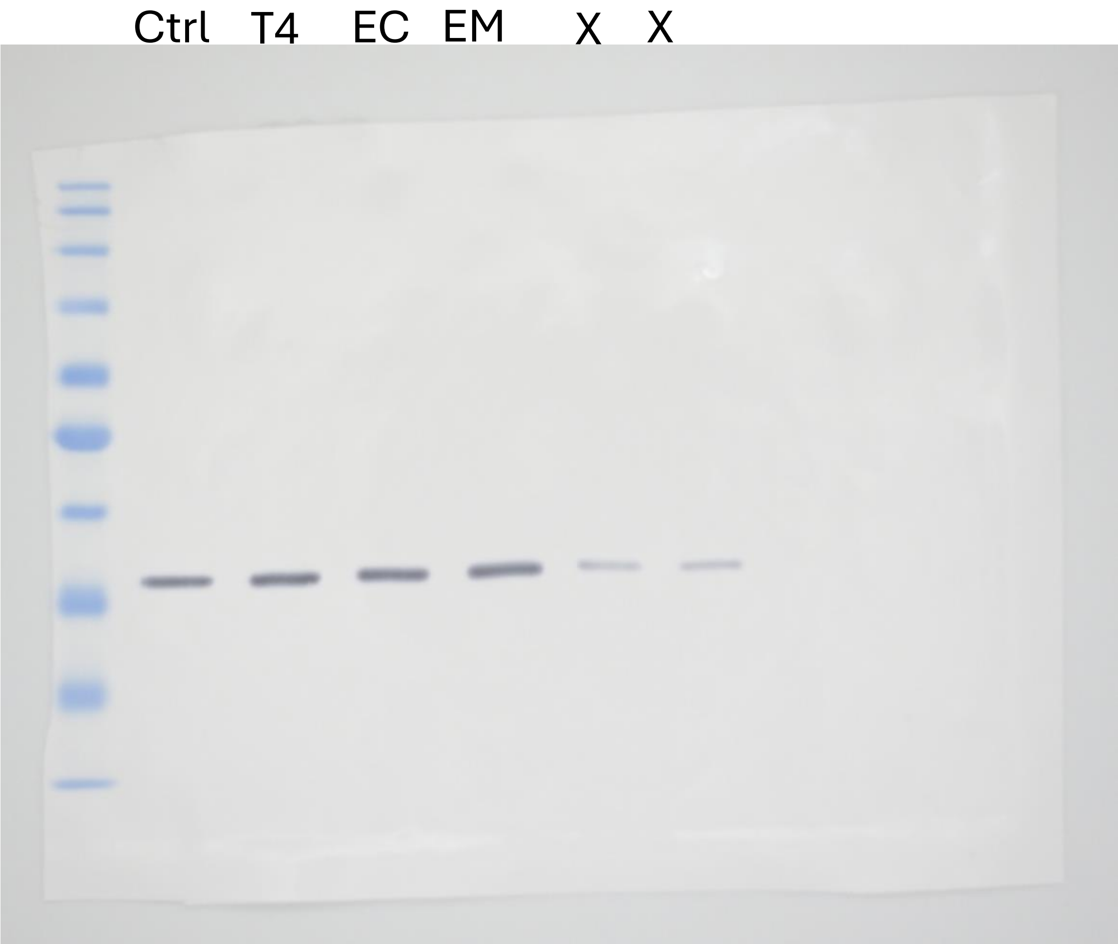

Figure 3A and B. PINK1 and beta actin

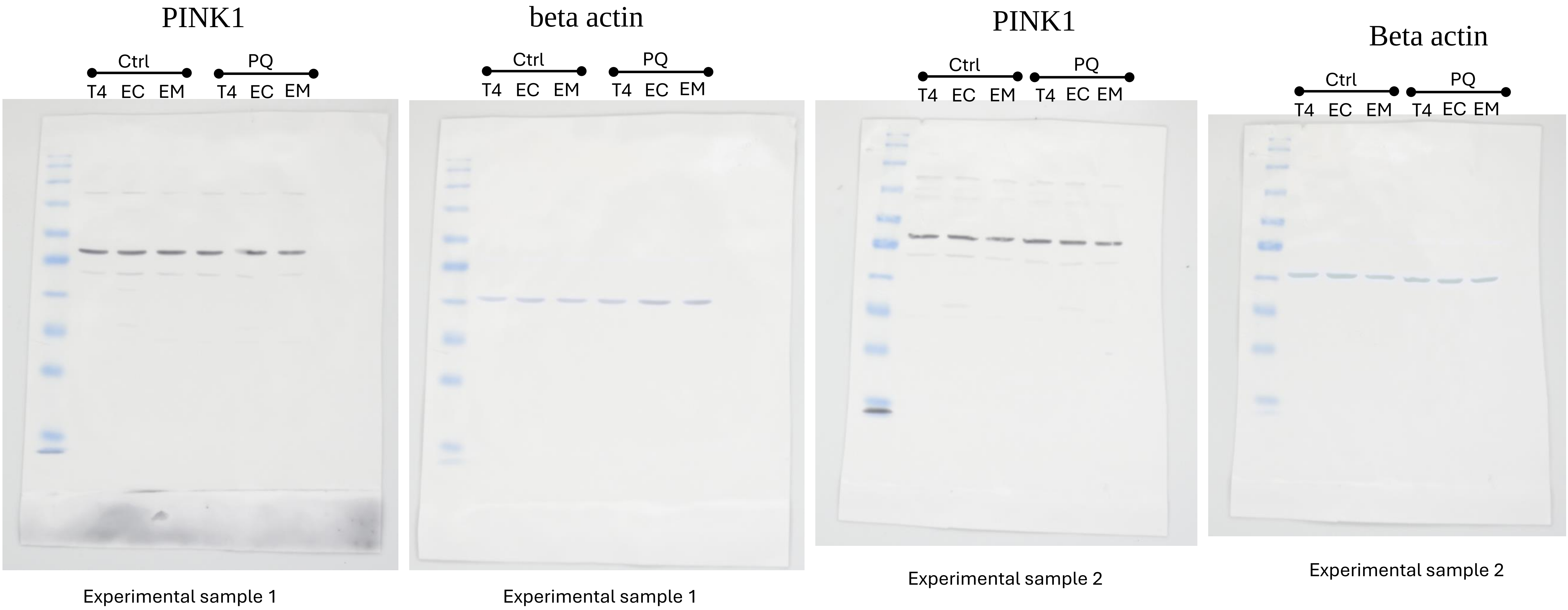

Figure 3A and B. PINK1 and beta actin

PINK1

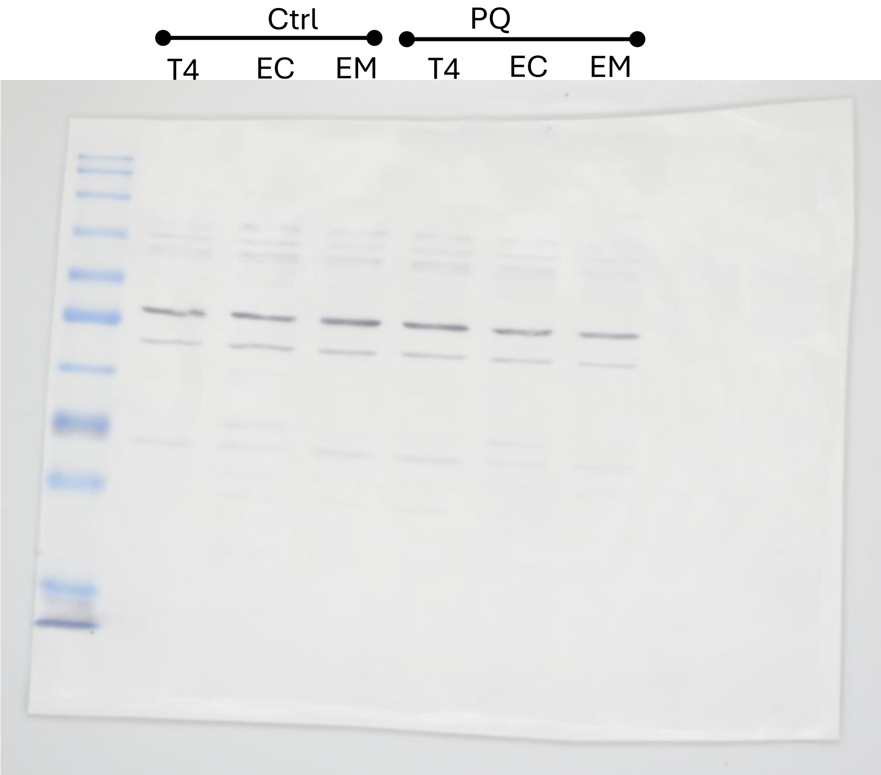

Experimental sample 3

Beta actin

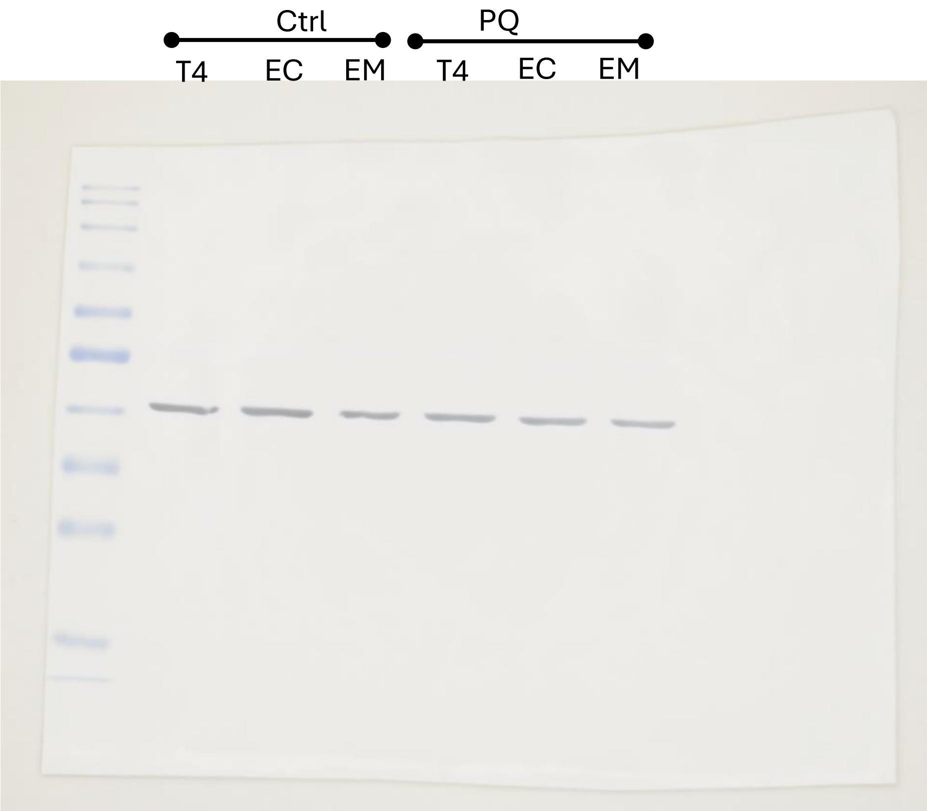

Experimental sample 3

Figure 3A and C. Parkin and beta actin

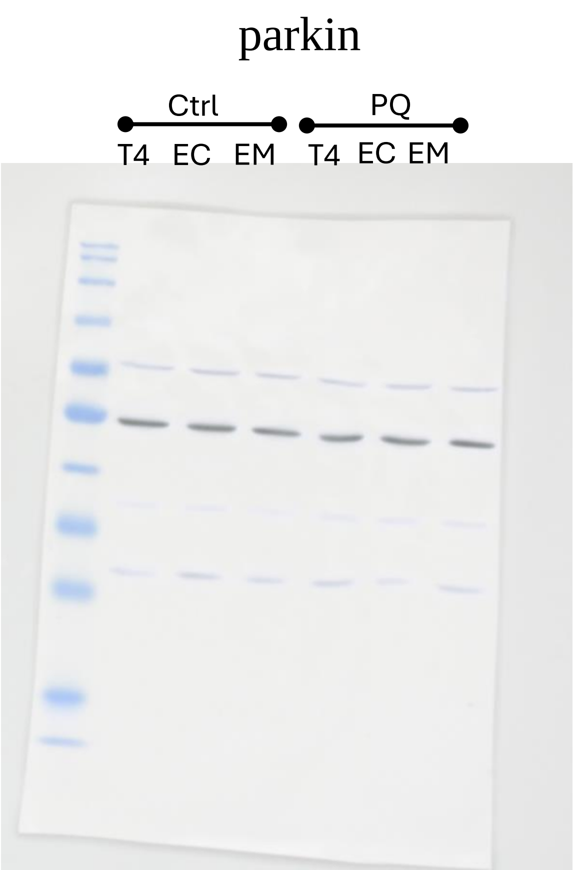

Experimental sample 1

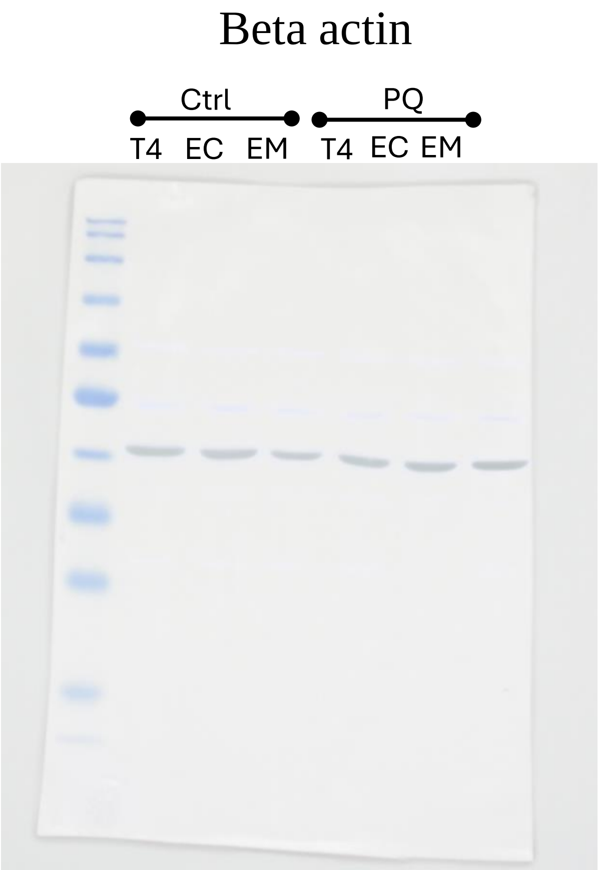

Experimental sample 1

Figure 3A and C. Parkin and beta actin

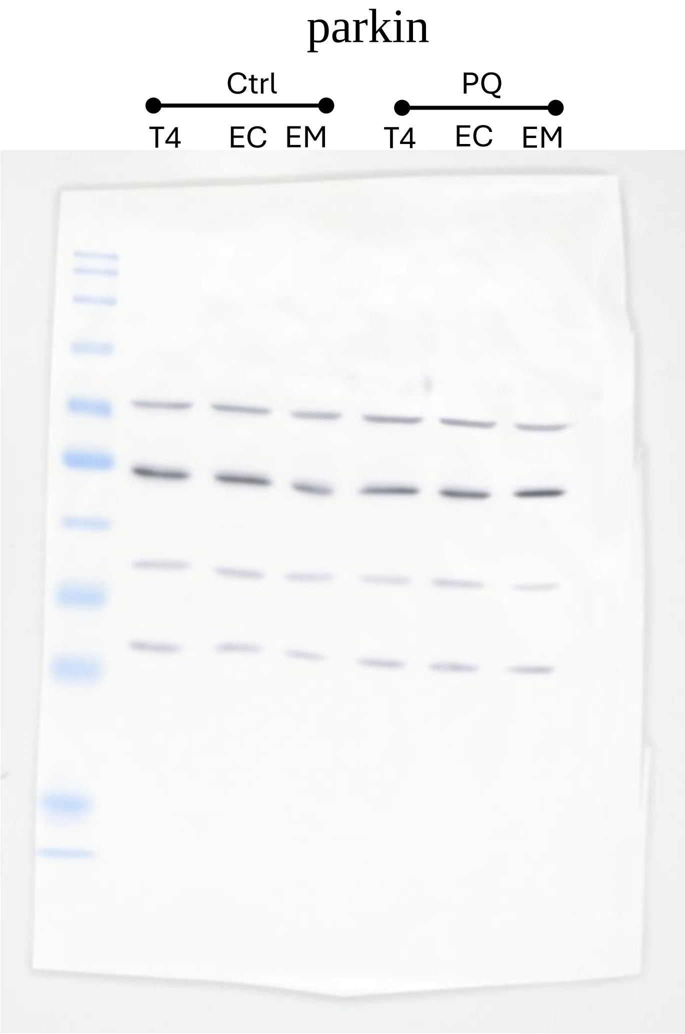

Experimental sample 2

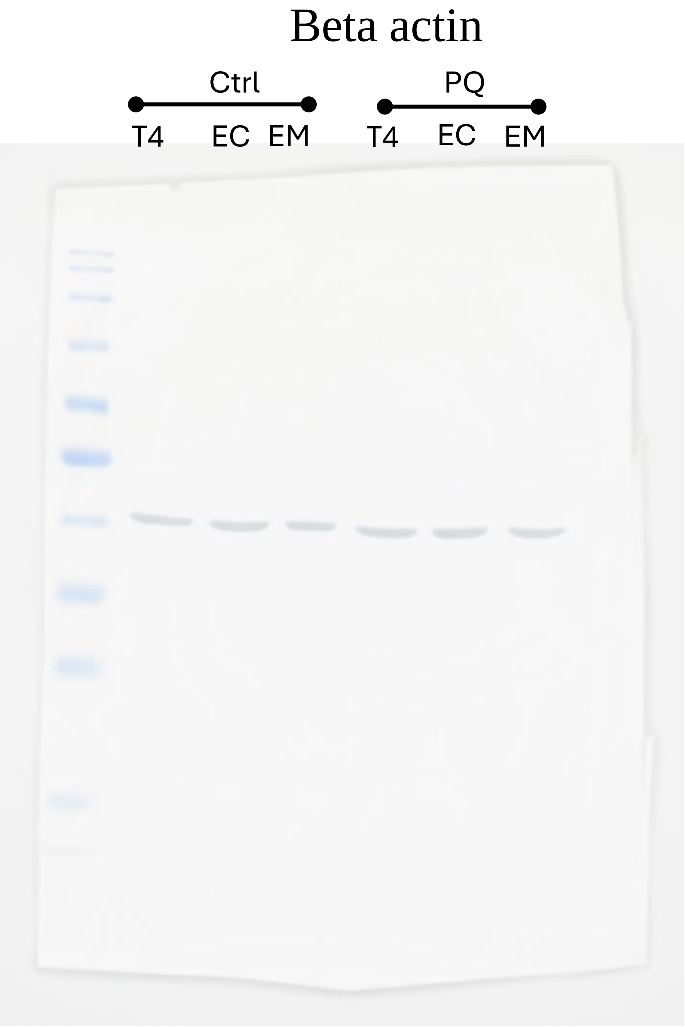

Experimental sample 2

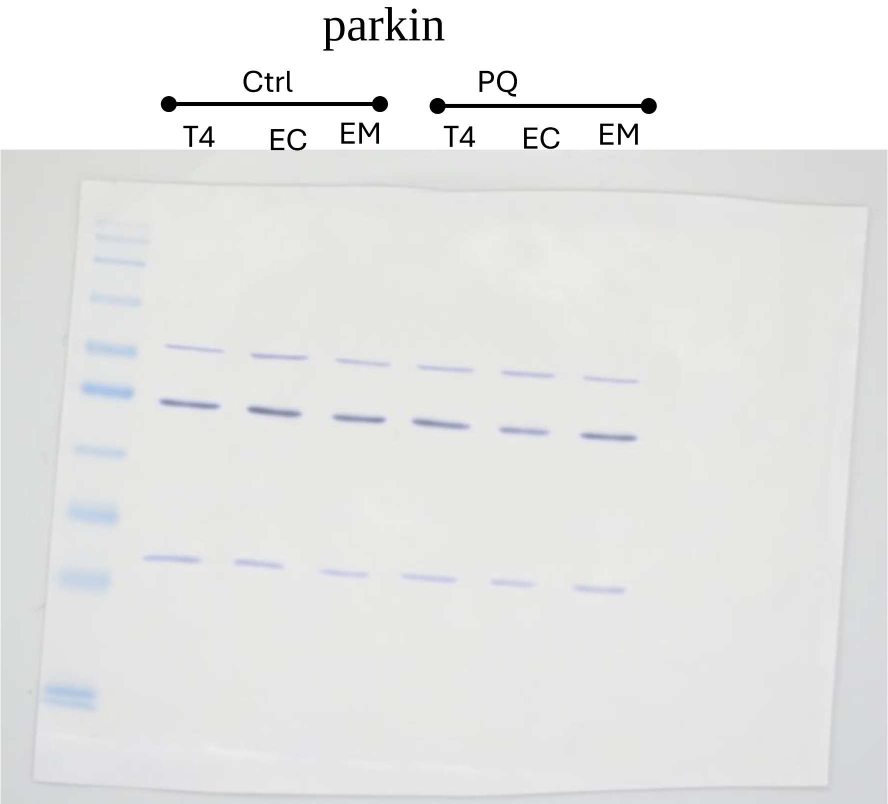

Experimental sample 3

Figure 3D and E. FKBP8 and beta actin

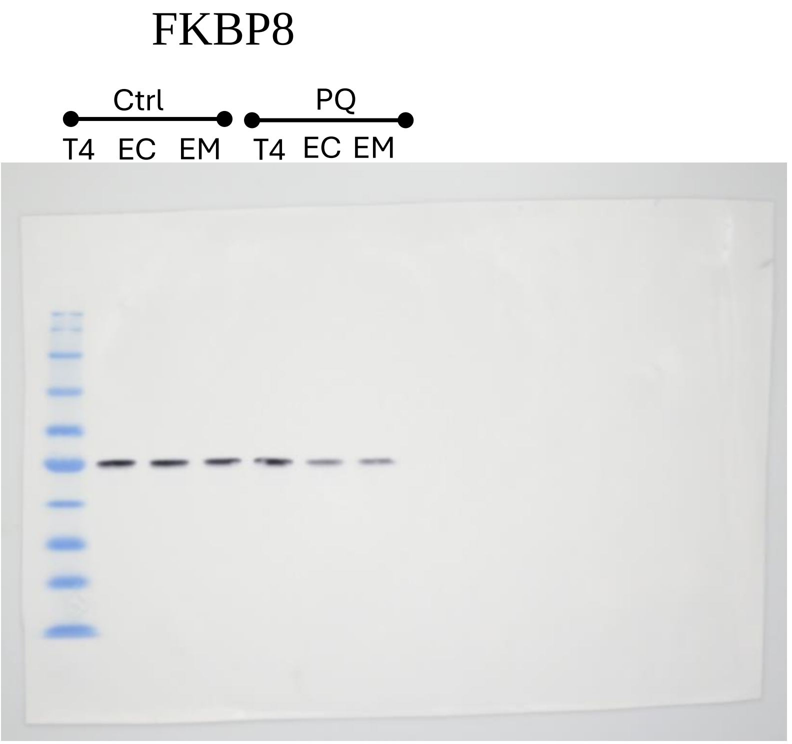

Experimental sample 1

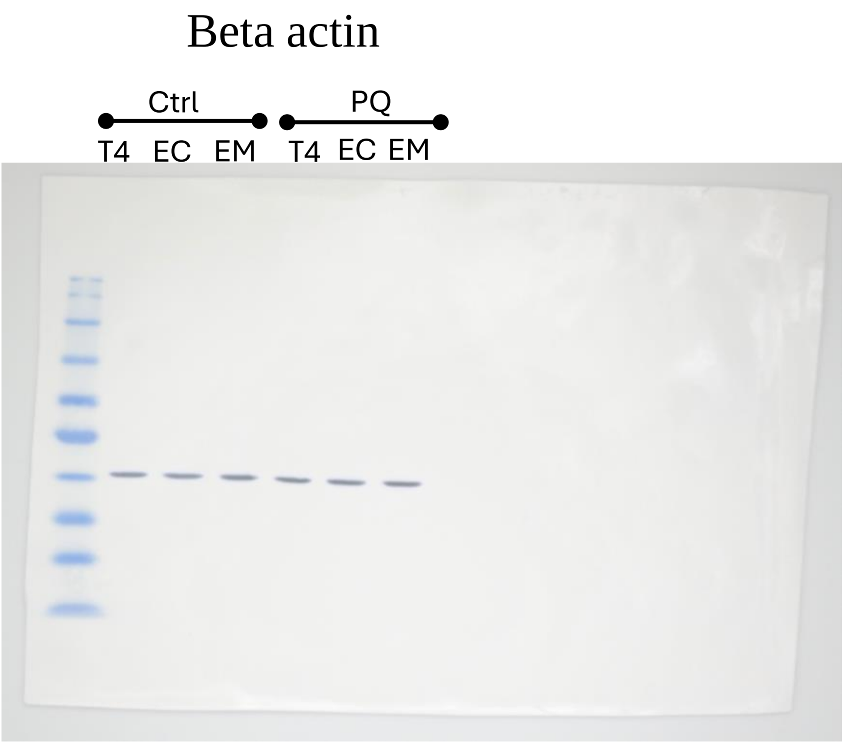

Experimental sample 1

Figure 3D and E. FKBP8 and beta actin

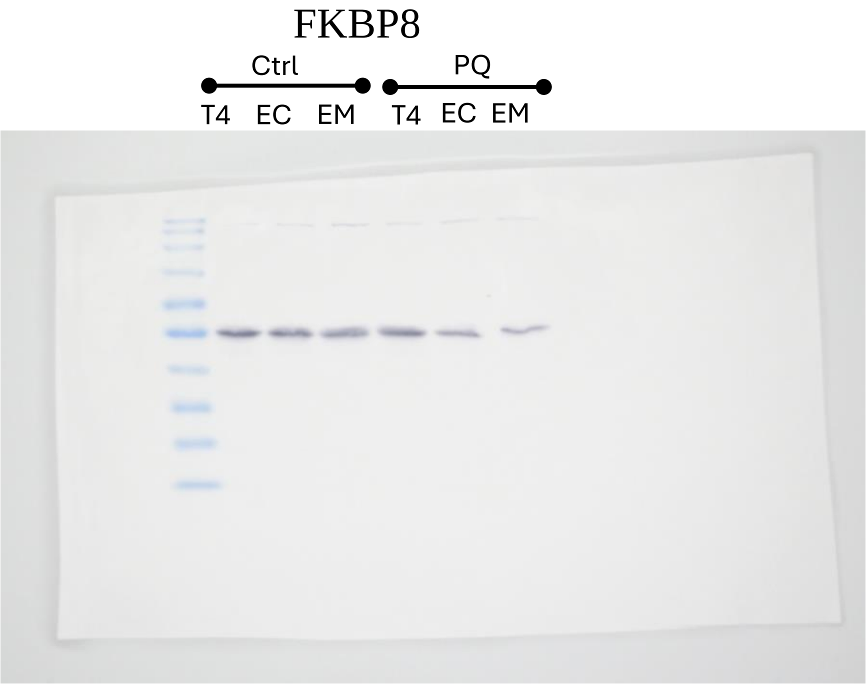

Experimental sample 2

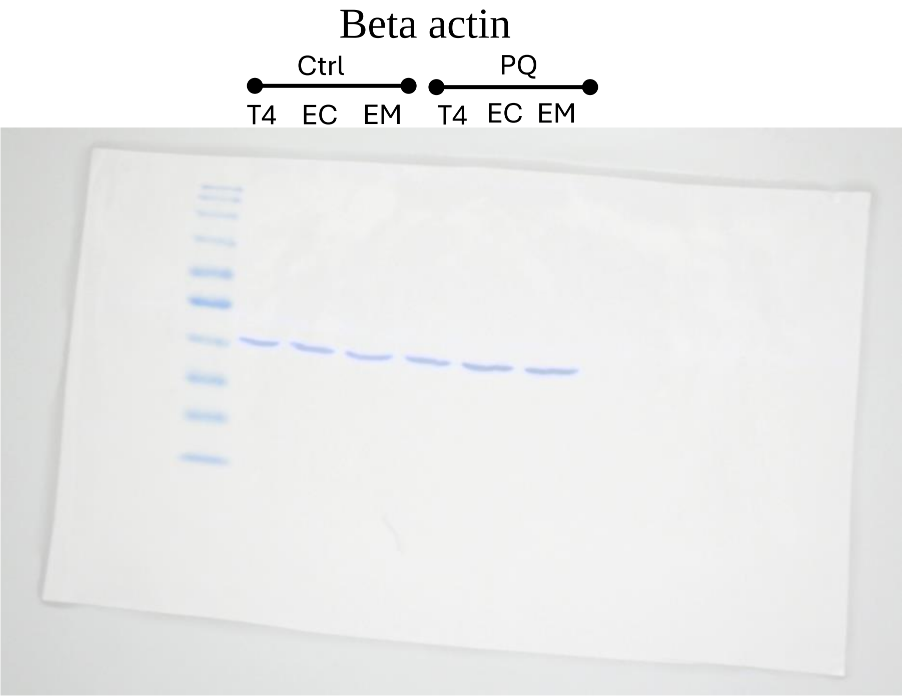

Experimental sample 2

Figure 3D and E. FKBP8 and beta actin

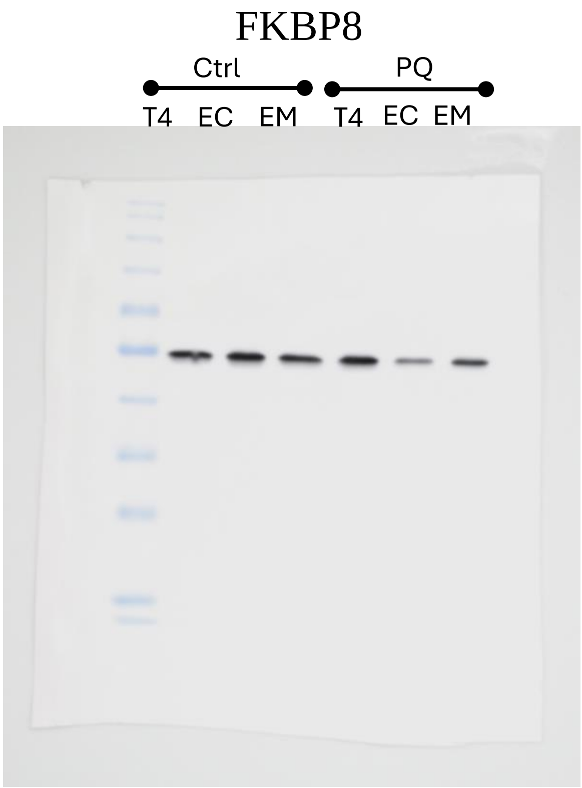

Experimental sample 3

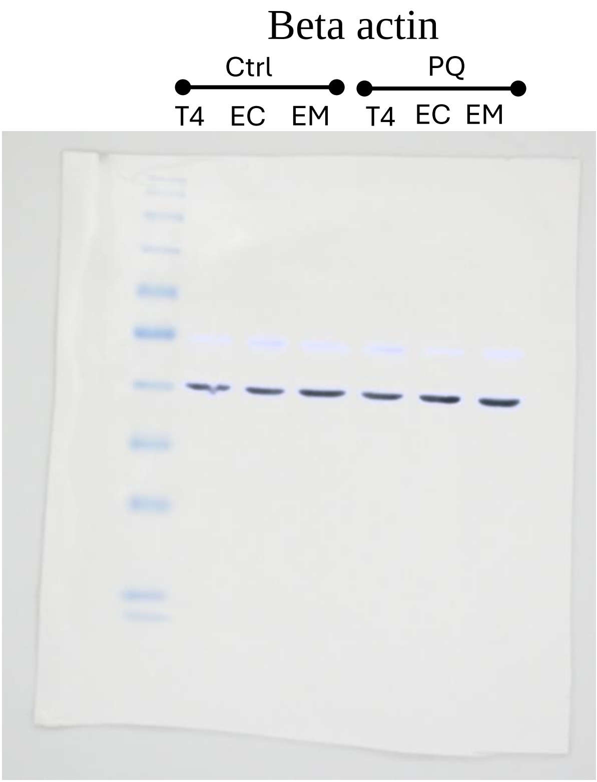

Experimental sample 3

Figure 3D and E. FKBP8 and beta actin

FKBP8

Ctrl PQ

T4 EC EM T4 EC EM

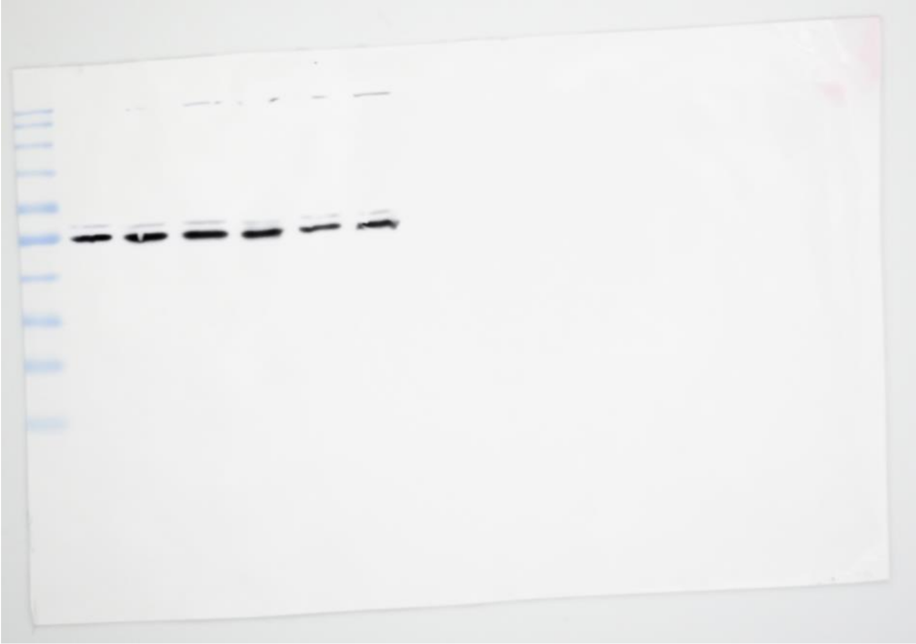

Experimental sample 4

Beta actin

Ctrl PQ

T4 EC EM T4 EC EM

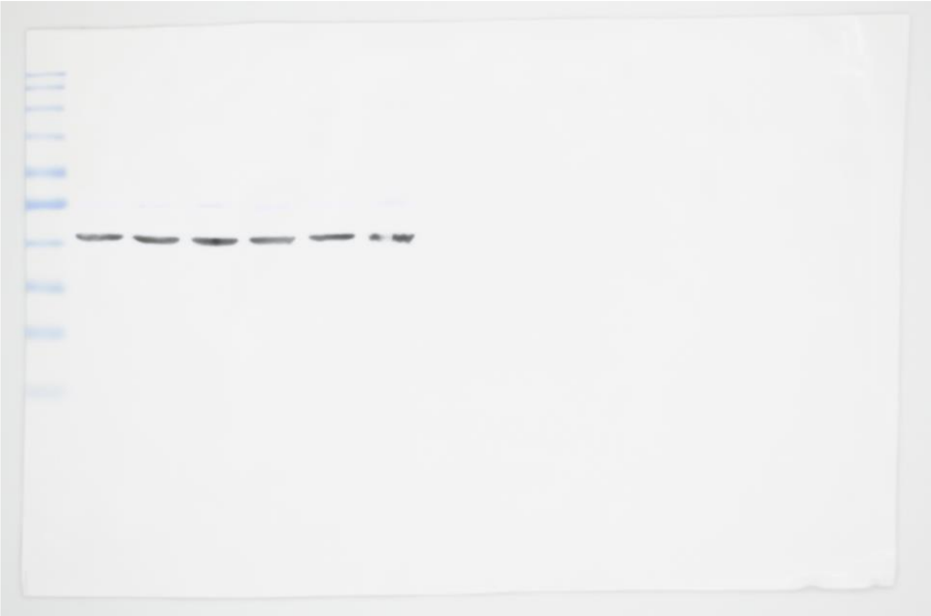

Experimental sample 4

Figure 3D and E. FKBP8 and beta actin

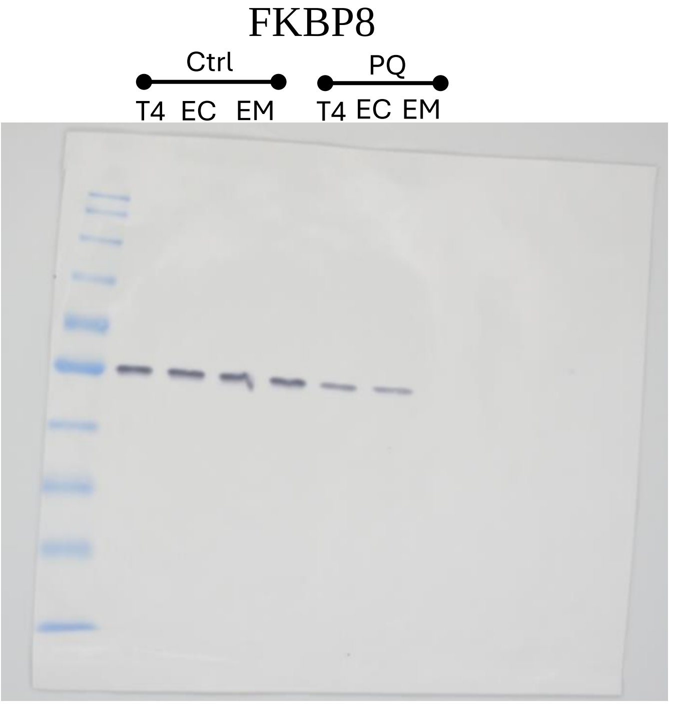

Experimental sample 5

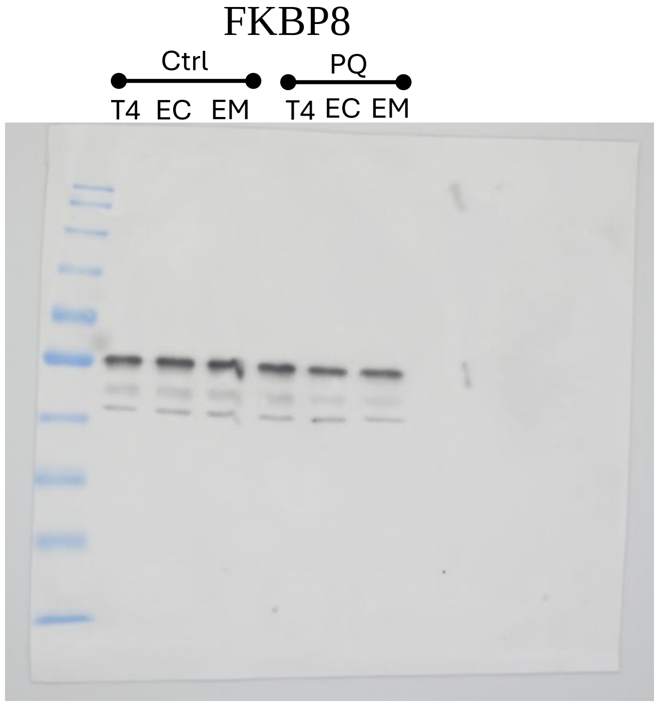

Experimental sample 5

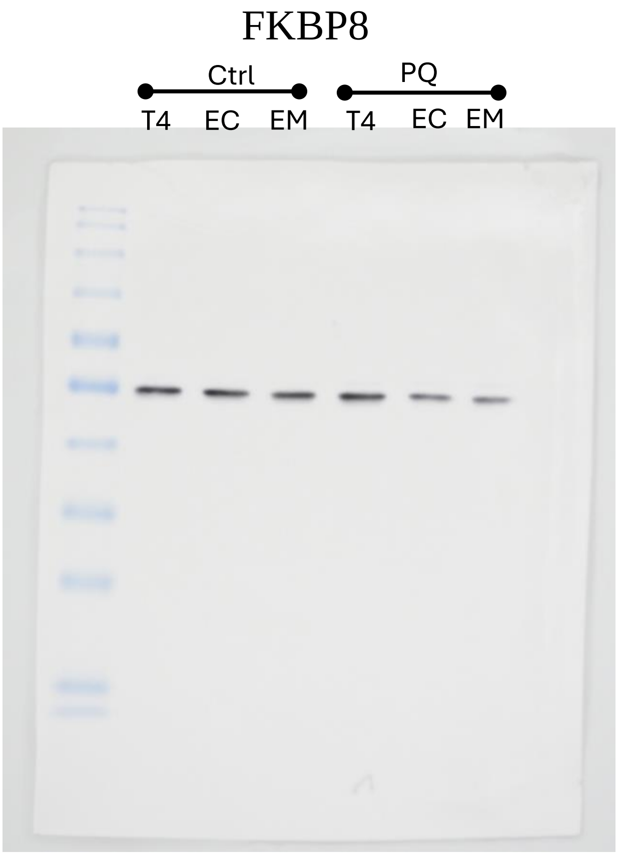
FKBP8
Ctrl
PQ
T4
EC
EM
T4
EC
EM

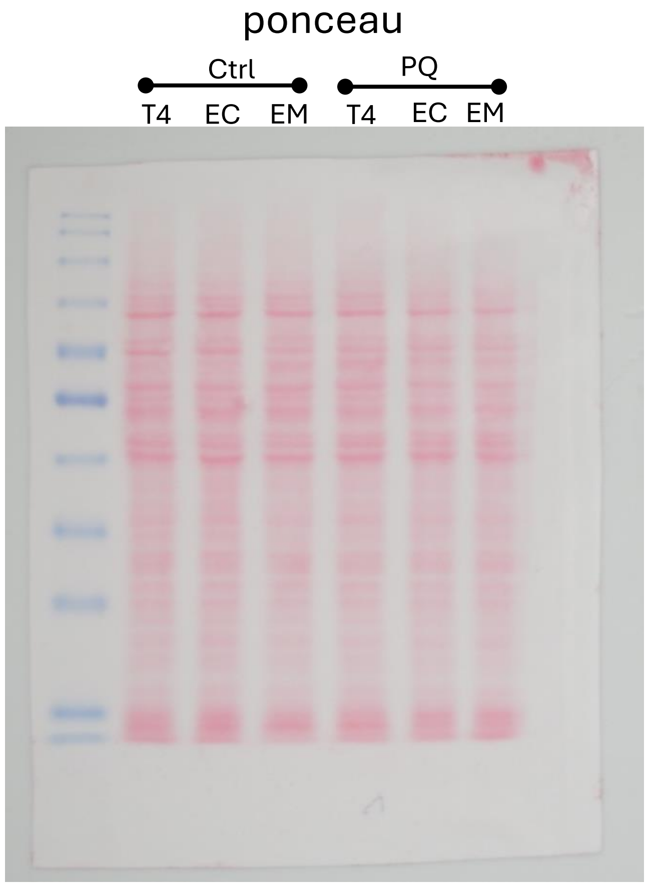
ponceau
Ctrl
PQ
T4
EC
EM
T4
EC
EM

Figure 3D and G. FUNDCI and beta actin

FUNDC1

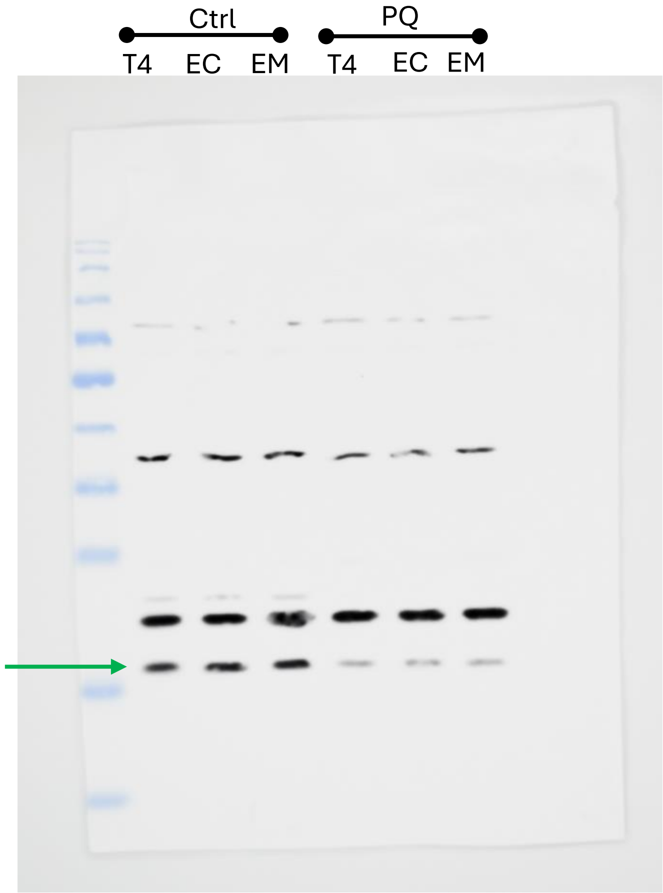

Experimental sample 1

Beta actin

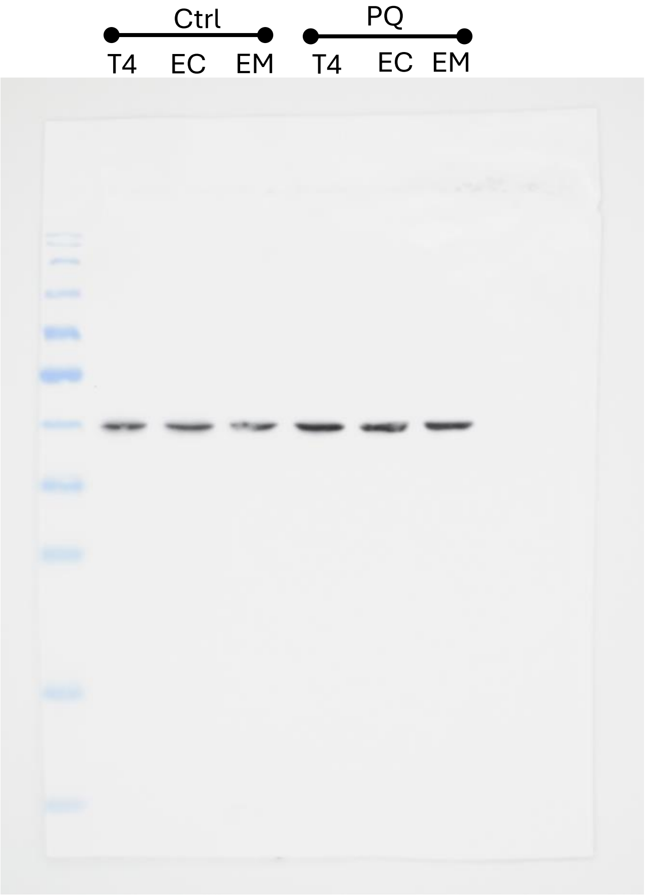

Experimental sample 1

Figure 3D and G. FUNDCI and beta actin

FUNDC1

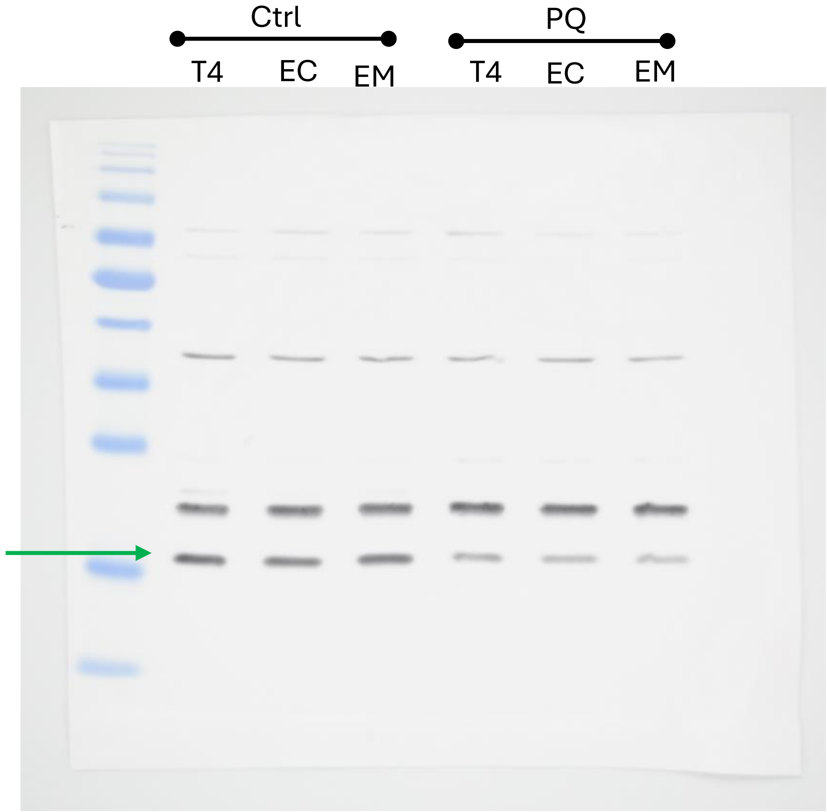

Experimental sample 2

Beta actin

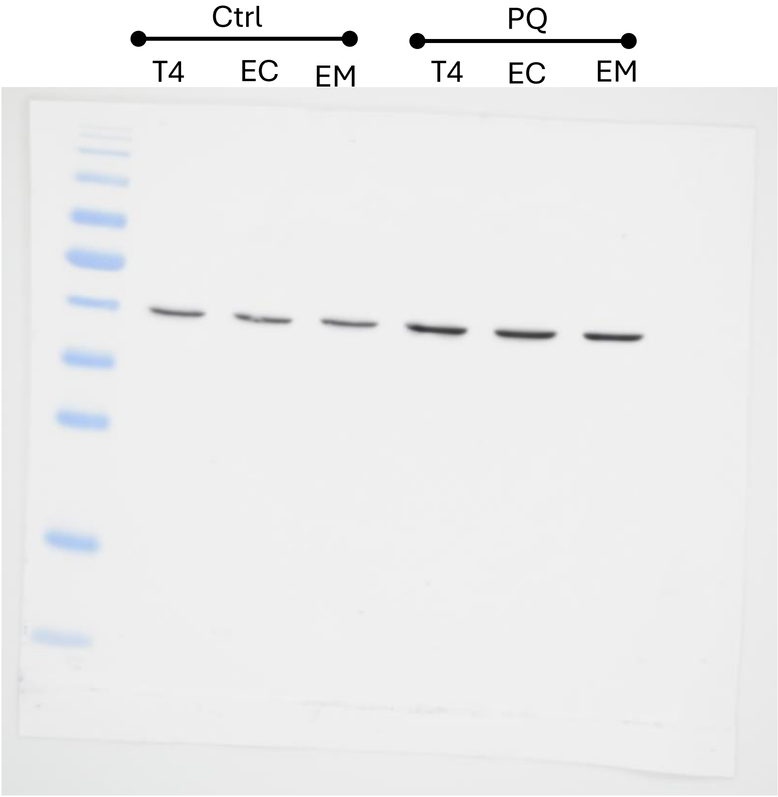

Experimental sample 2

Figure 3D and G. FUNDCI and beta actin

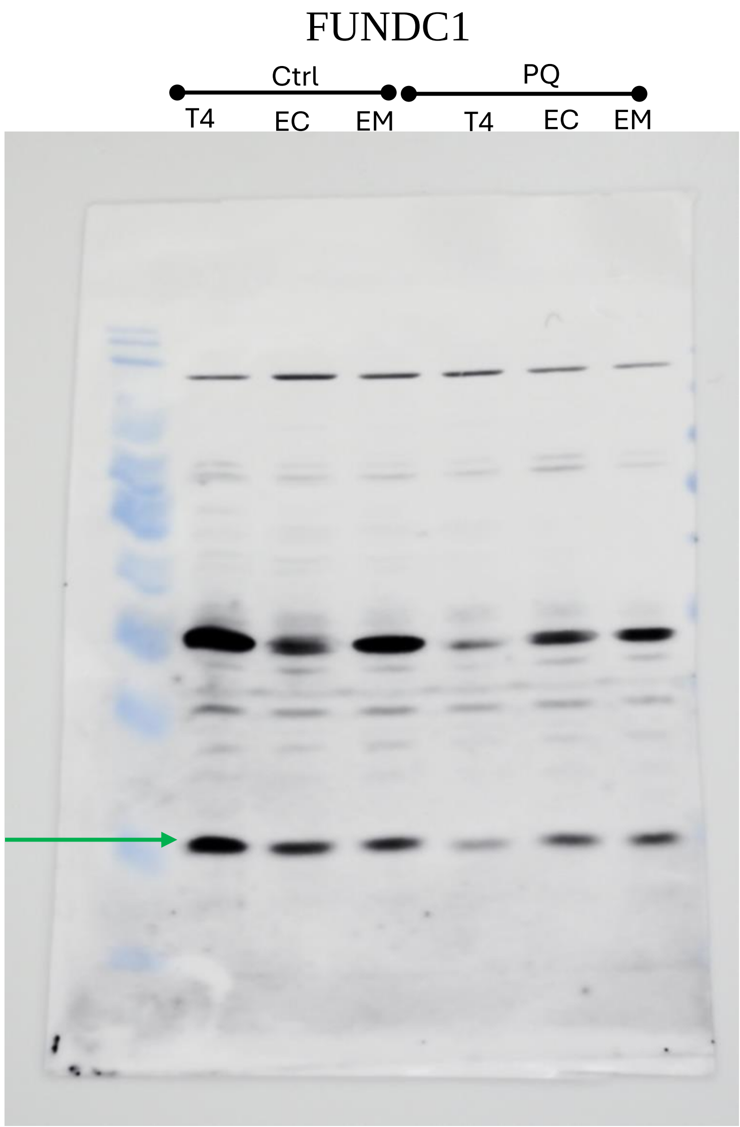

Experimental sample 3

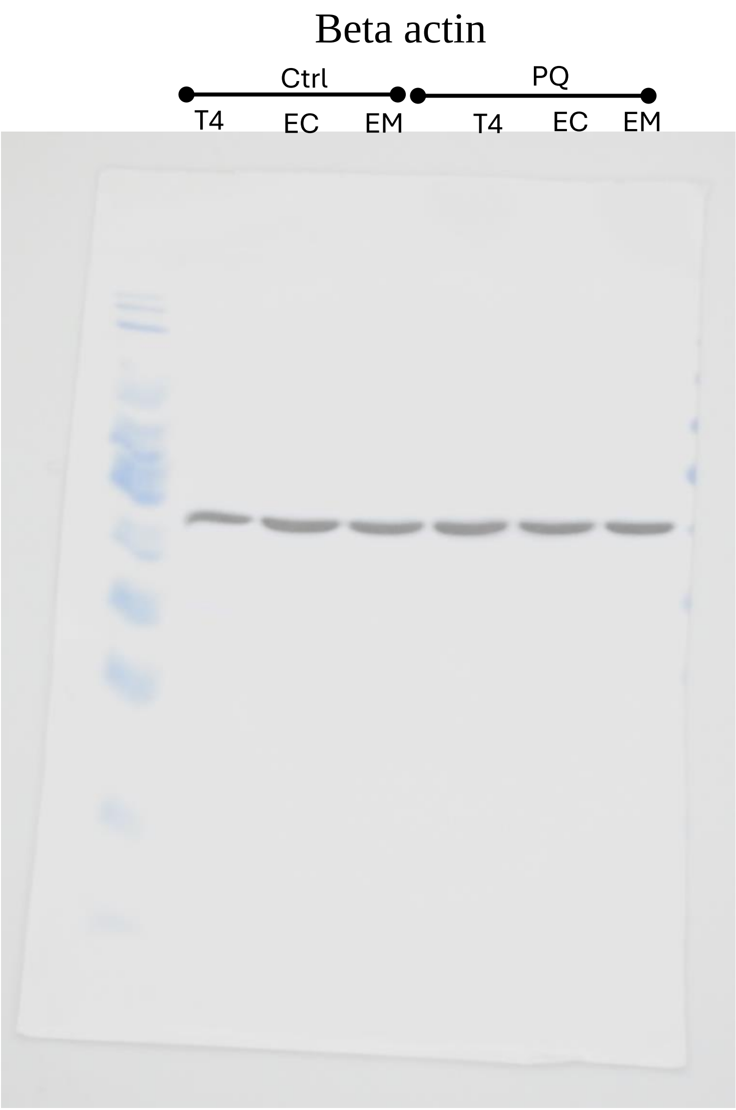

Experimental sample 3

Figure 3D and G. FUNDCI and beta actin

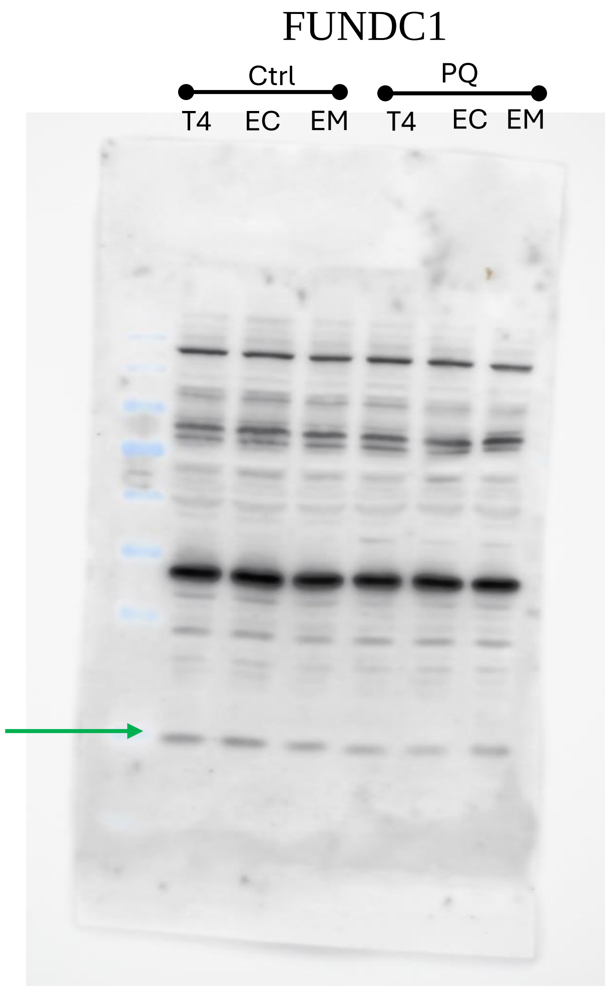

Experimental sample 4

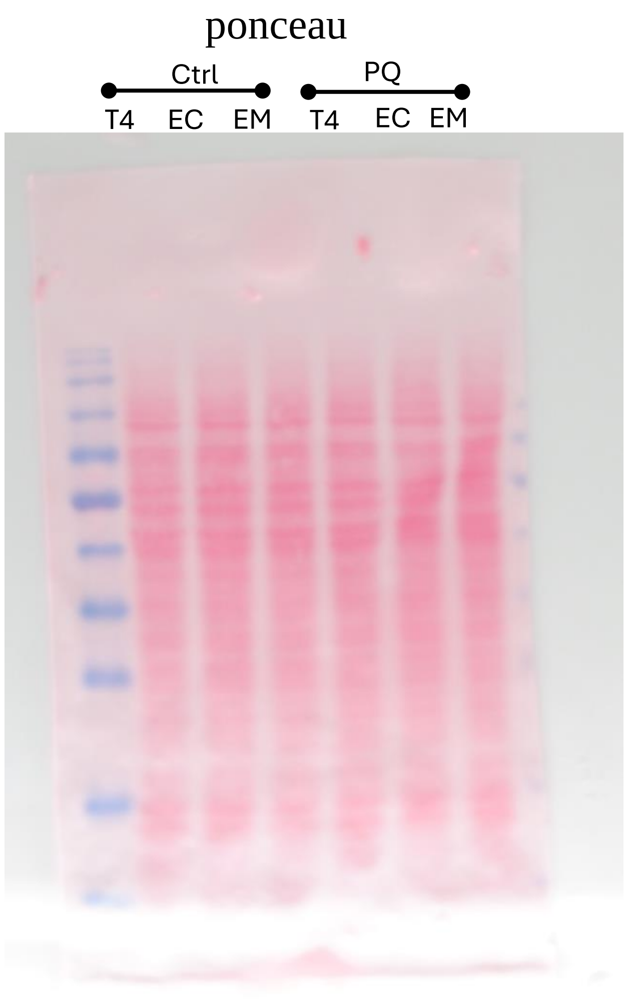

Experimental sample 4

Figure 3D and H. BNIP3 and beta actin

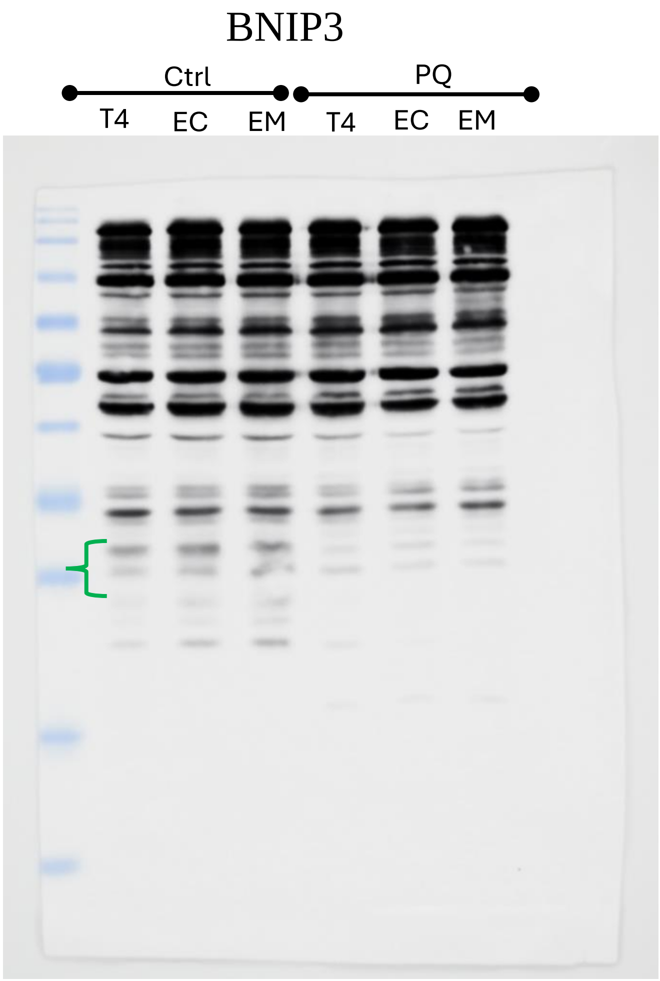

Experimental sample 1

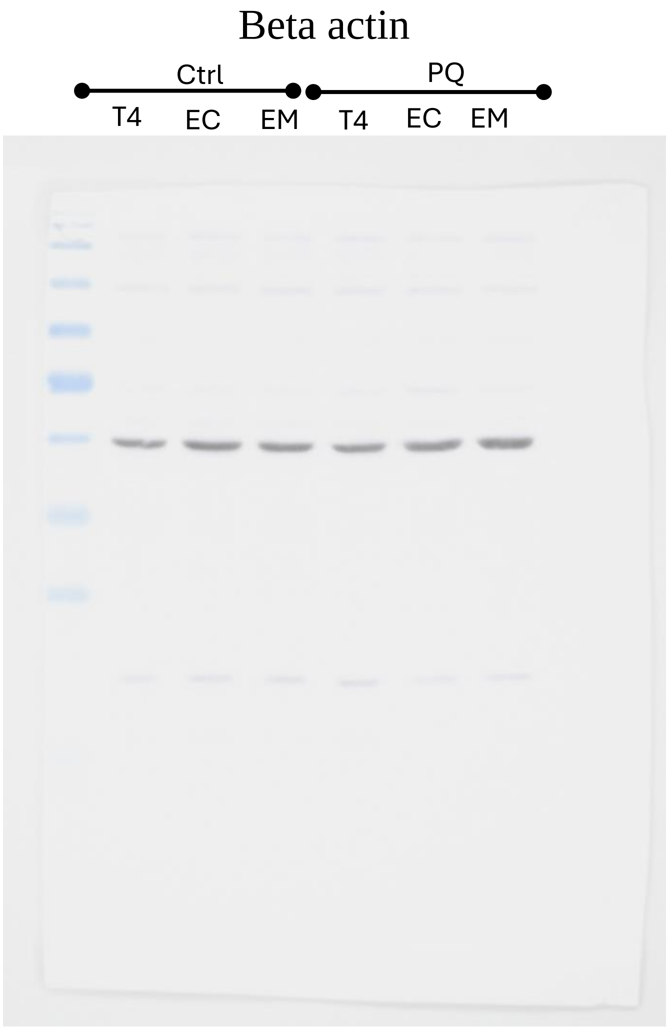

Experimental sample 1

Figure 3D and H. BNIP3 and beta actin

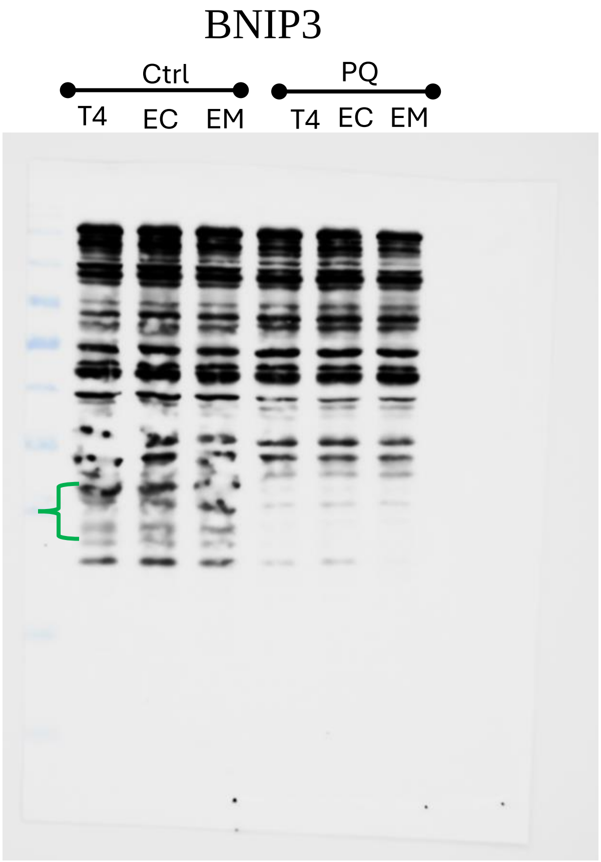

Experimental sample 2

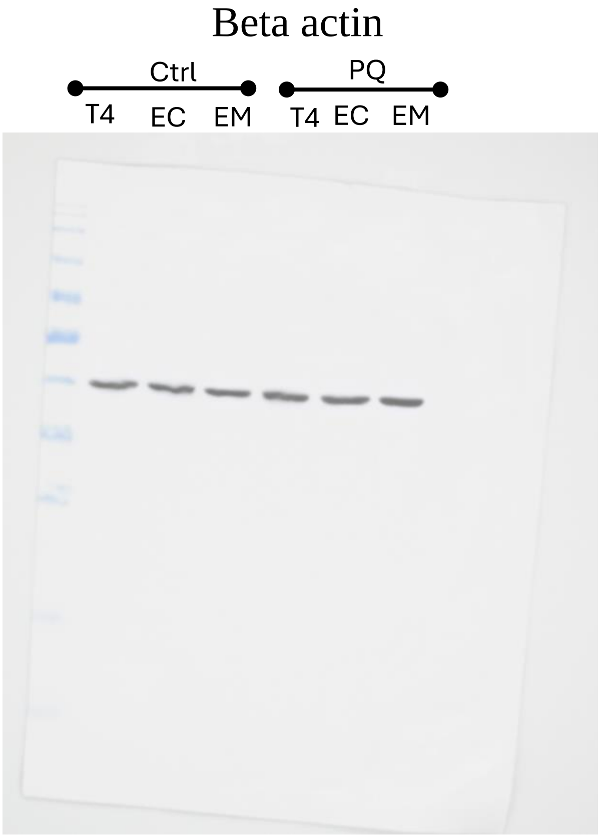

Experimental sample 2

Figure 3D and H. BNIP3 and beta actin

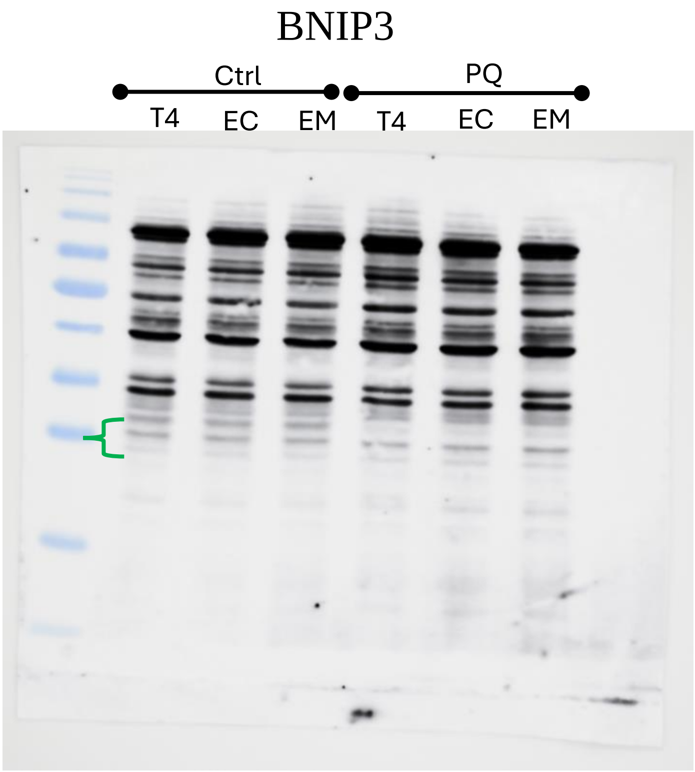

Experimental sample 3

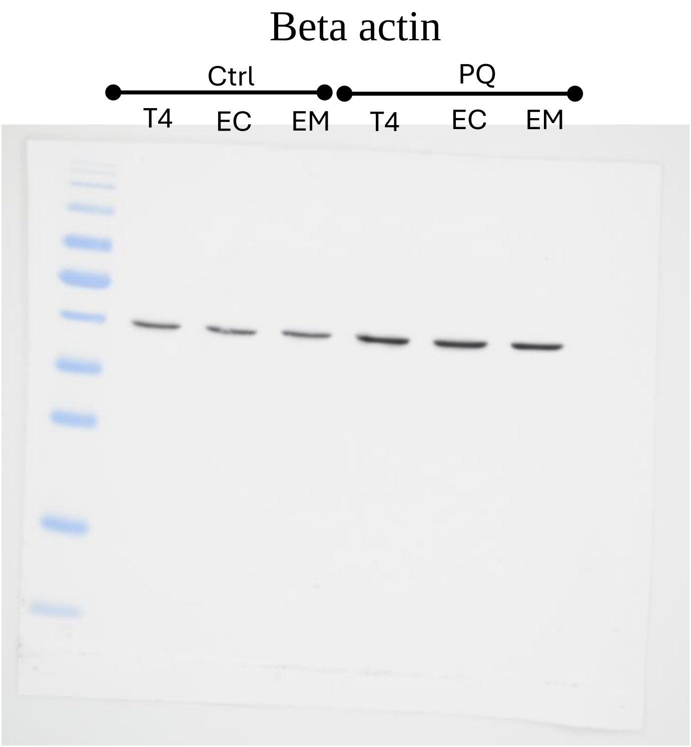

Experimental sample 3

Figure 3D and H. BNIP3 and beta actin

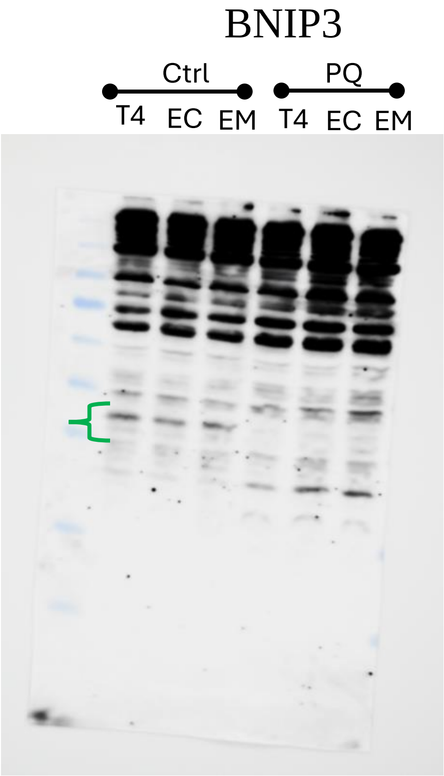

Experimental sample 4

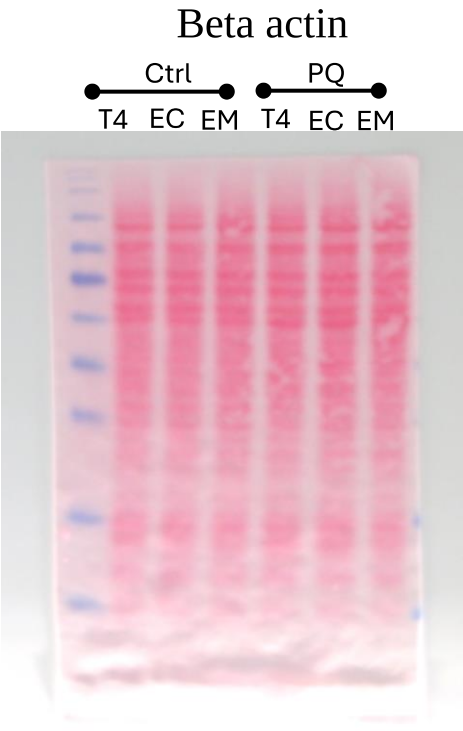

Experimental sample 4

Figure 3F

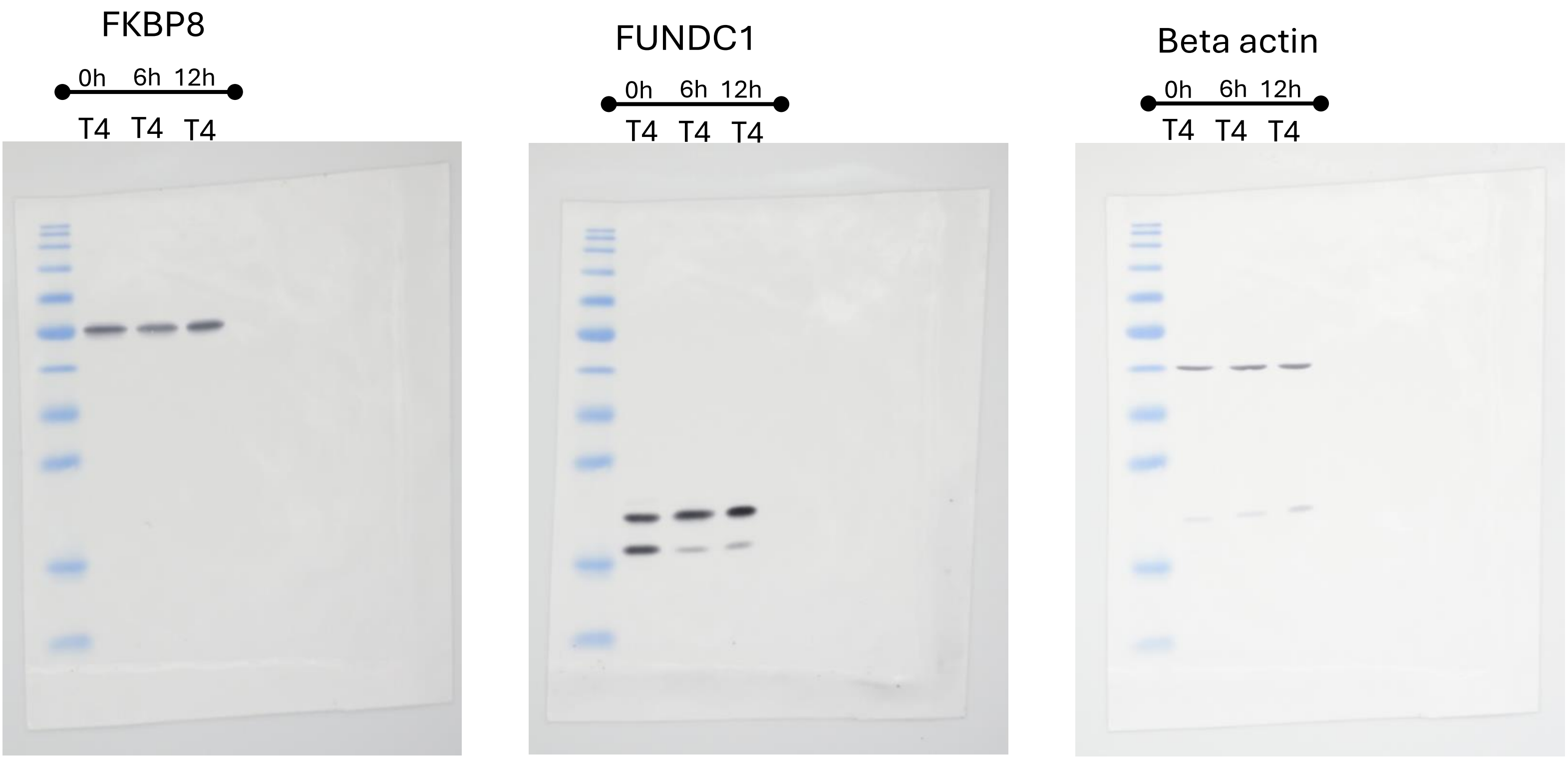
FKBP8
0h 6h 12h
T4 T4 T4
FUNDC1
0h 6h 12h
T4 T4 T4
Beta actin
0h 6h 12h
T4 T4 T4

Figure 4. CO-IP of tau and FKBP8

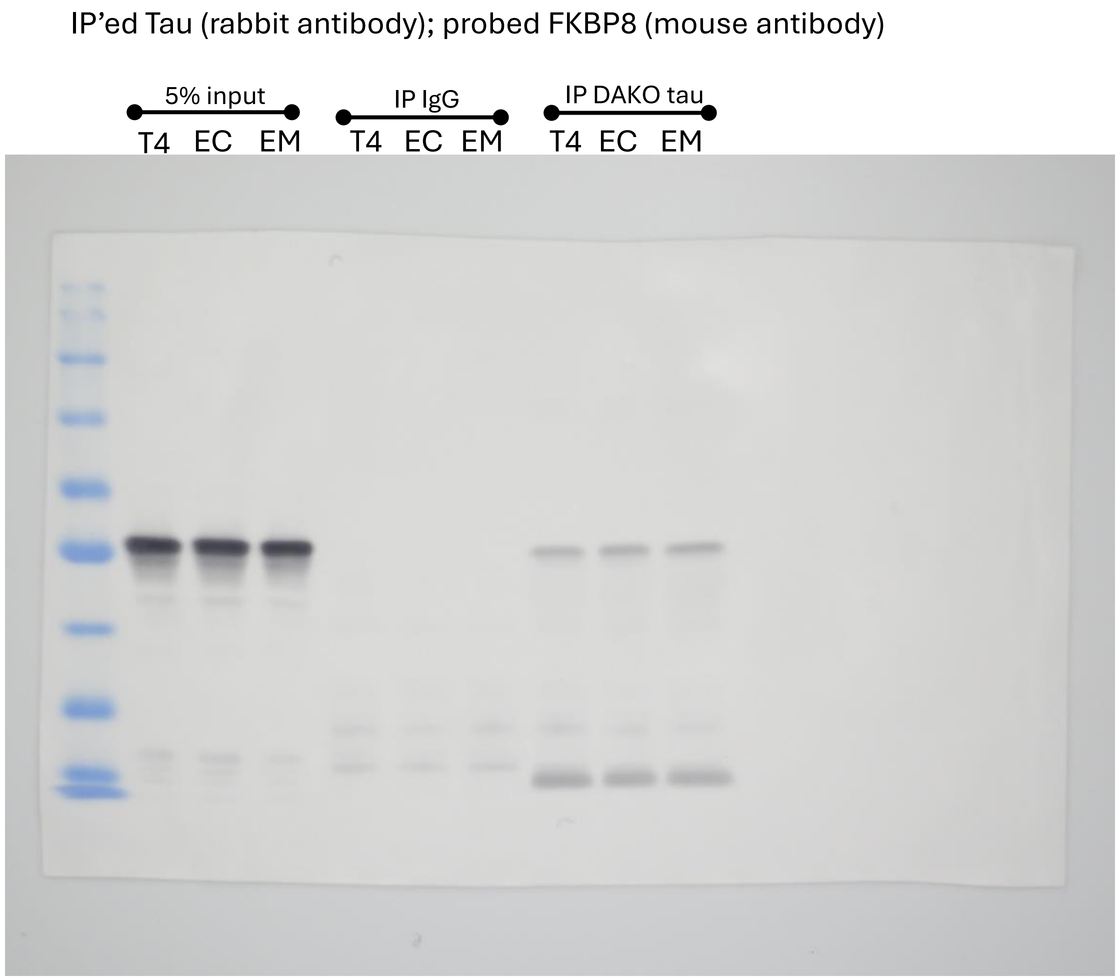

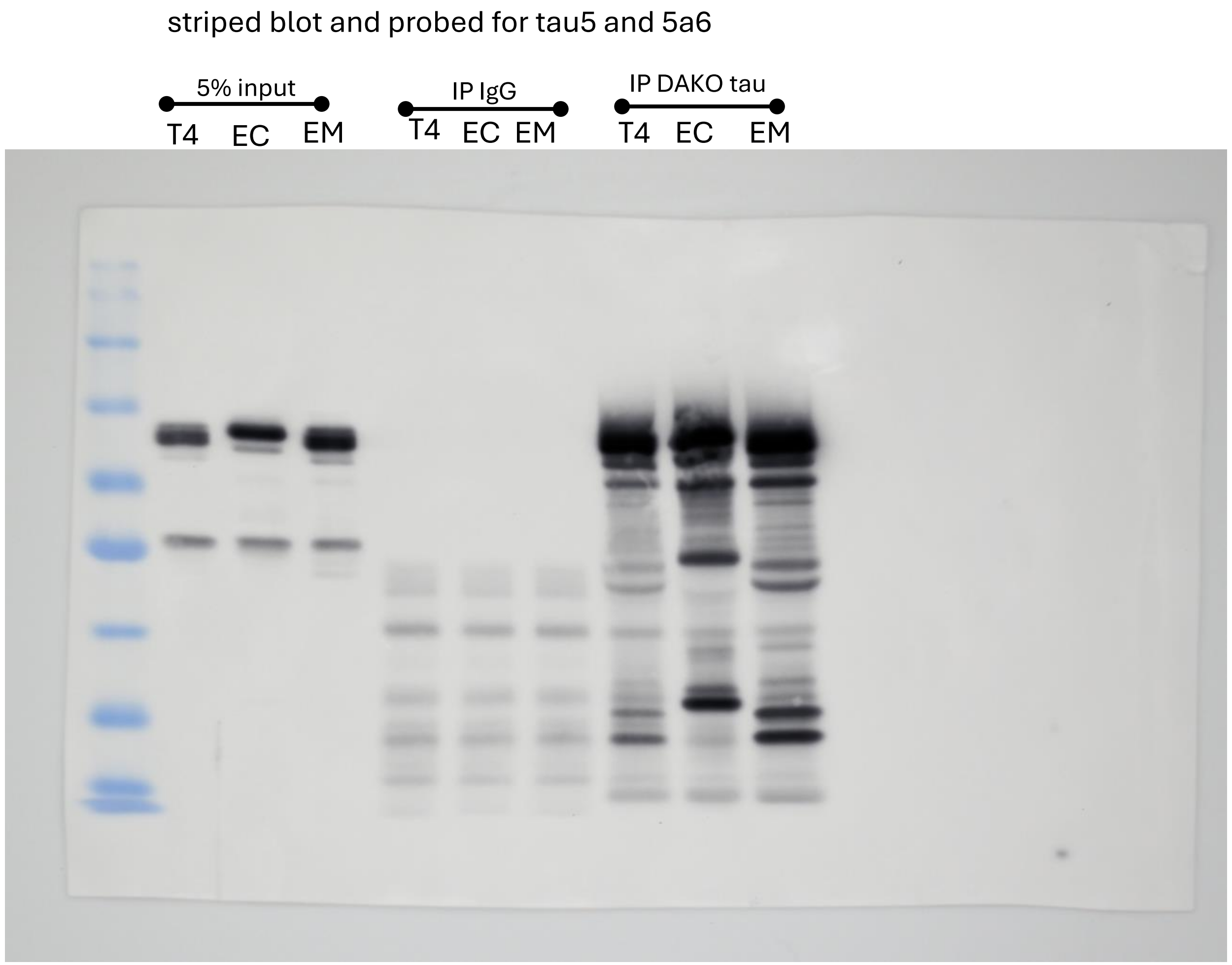

Figure 5D, E and F

FKBP8

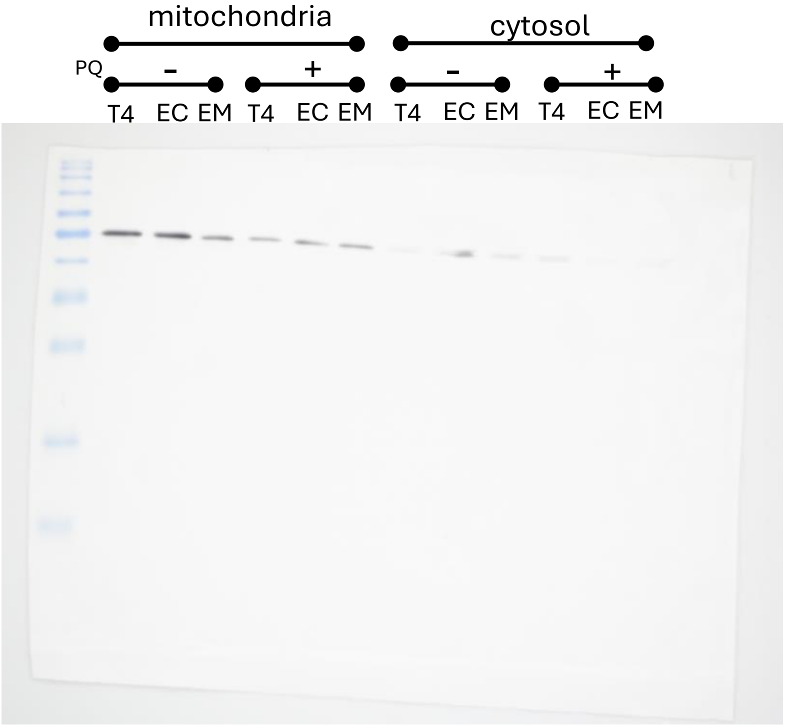

Experimental sample 1

TOM20

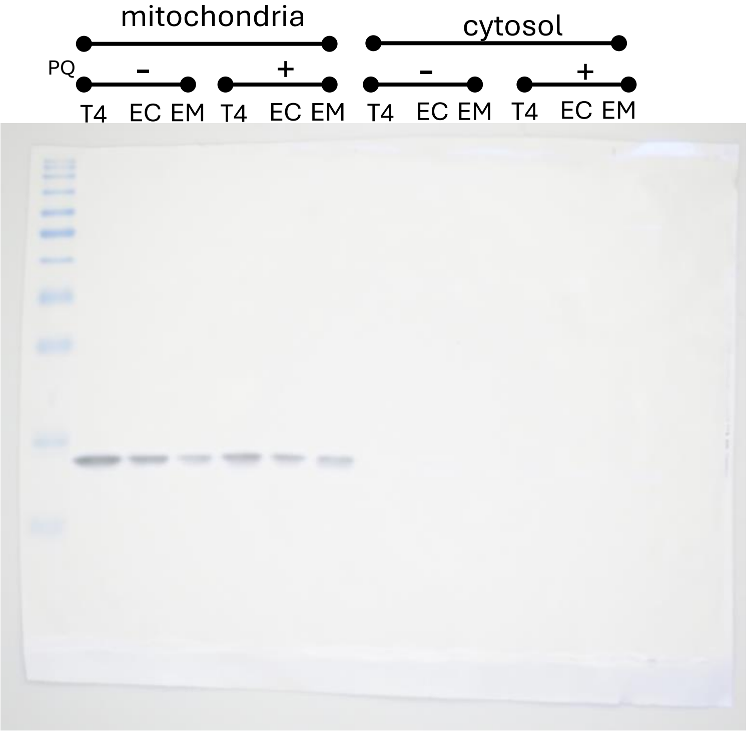

Experimental sample 1

Beta tubulin

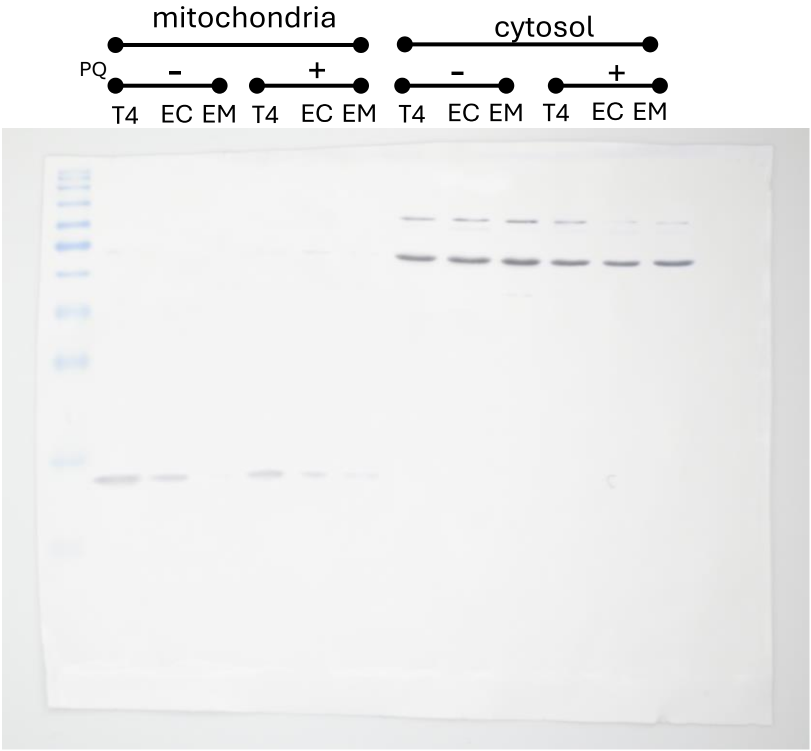

Experimental sample 1

Figure 5D, E and F

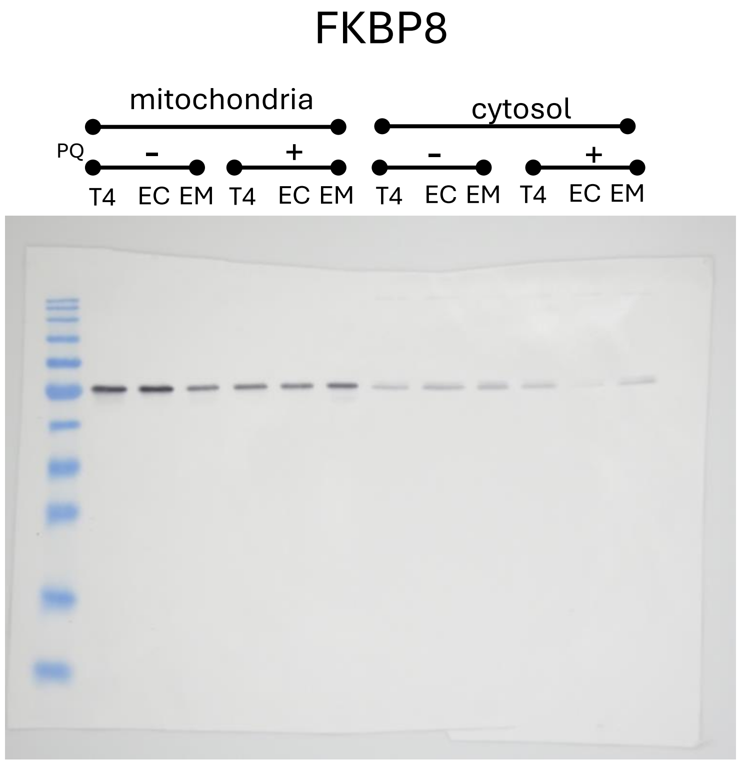

Experimental sample 2

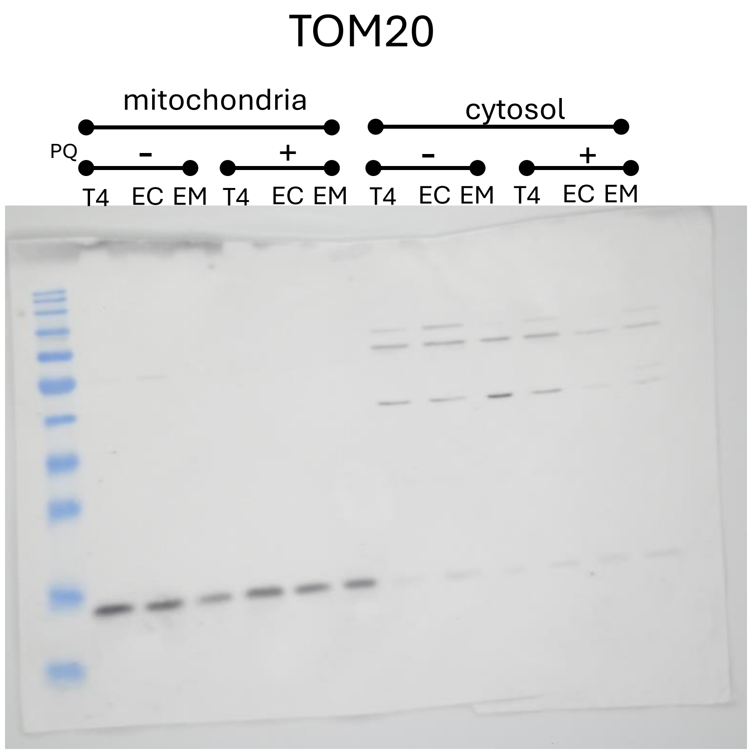

Experimental sample 2

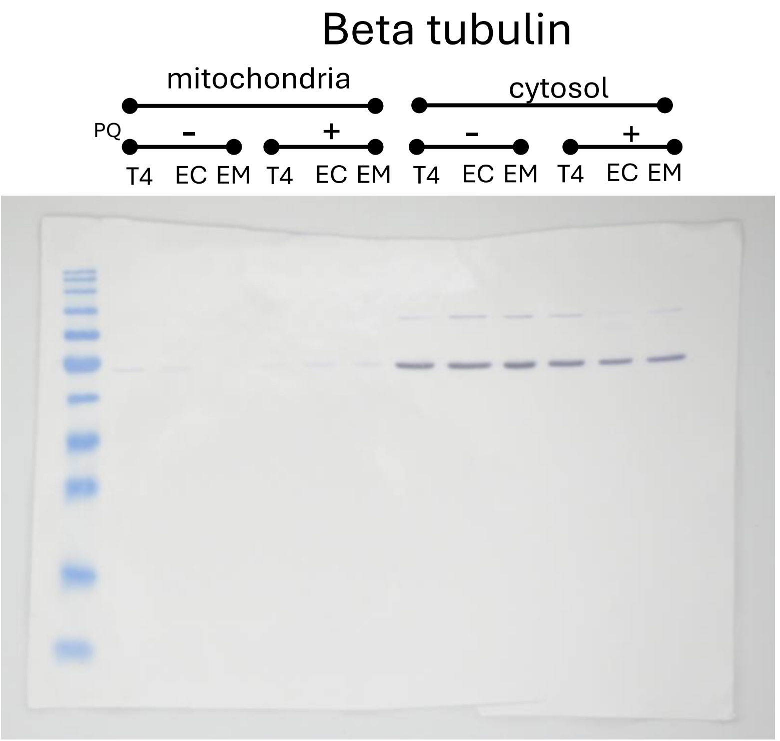

Experimental sample 2

Figure 5D, E and F

FKBP8

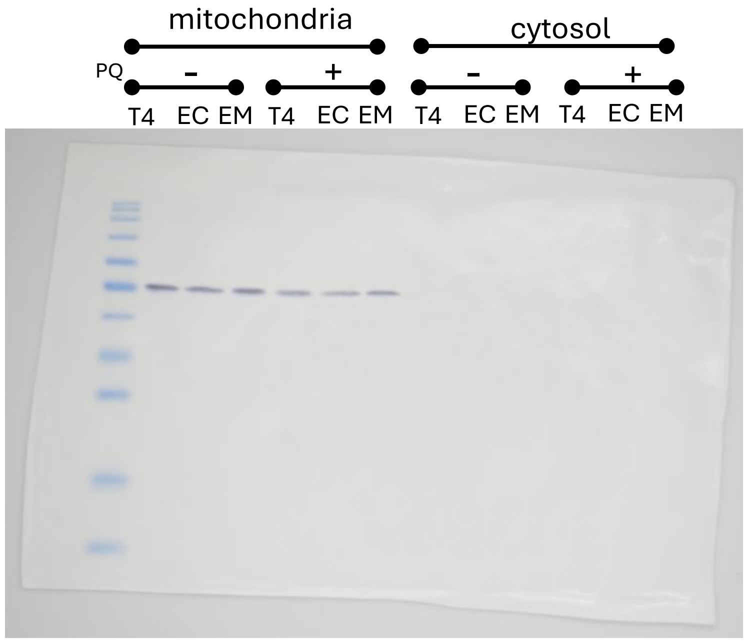

Experimental sample 3

TOM20

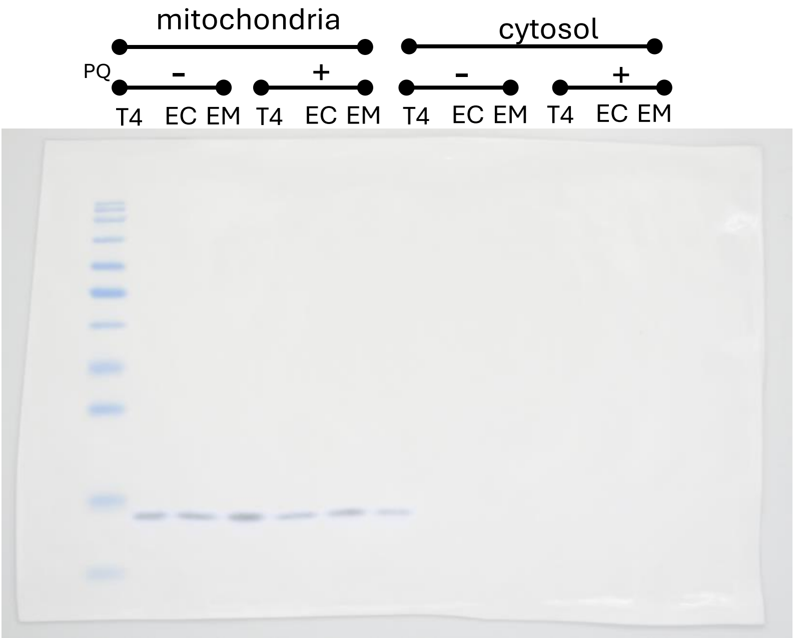

Experimental sample 3

Beta tubulin

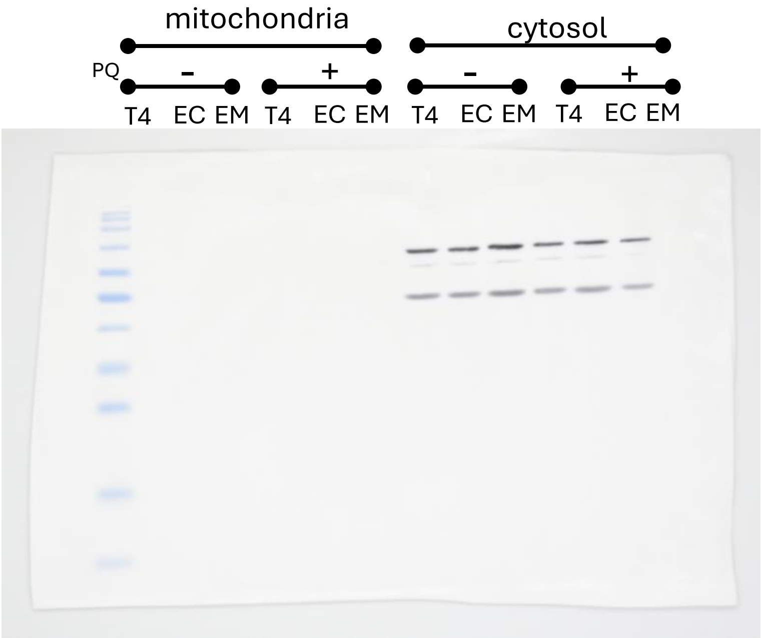

Experimental sample 3

Figure 6A. scramble vs shFKBP8

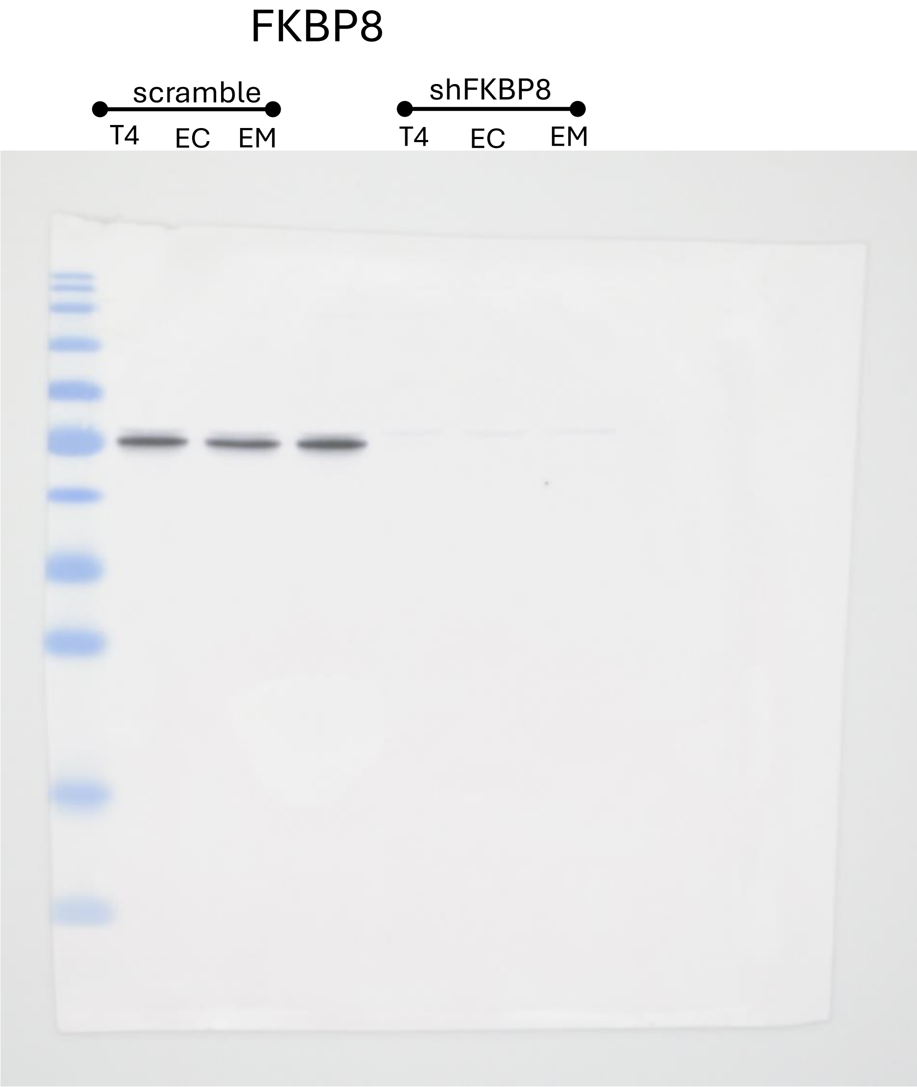

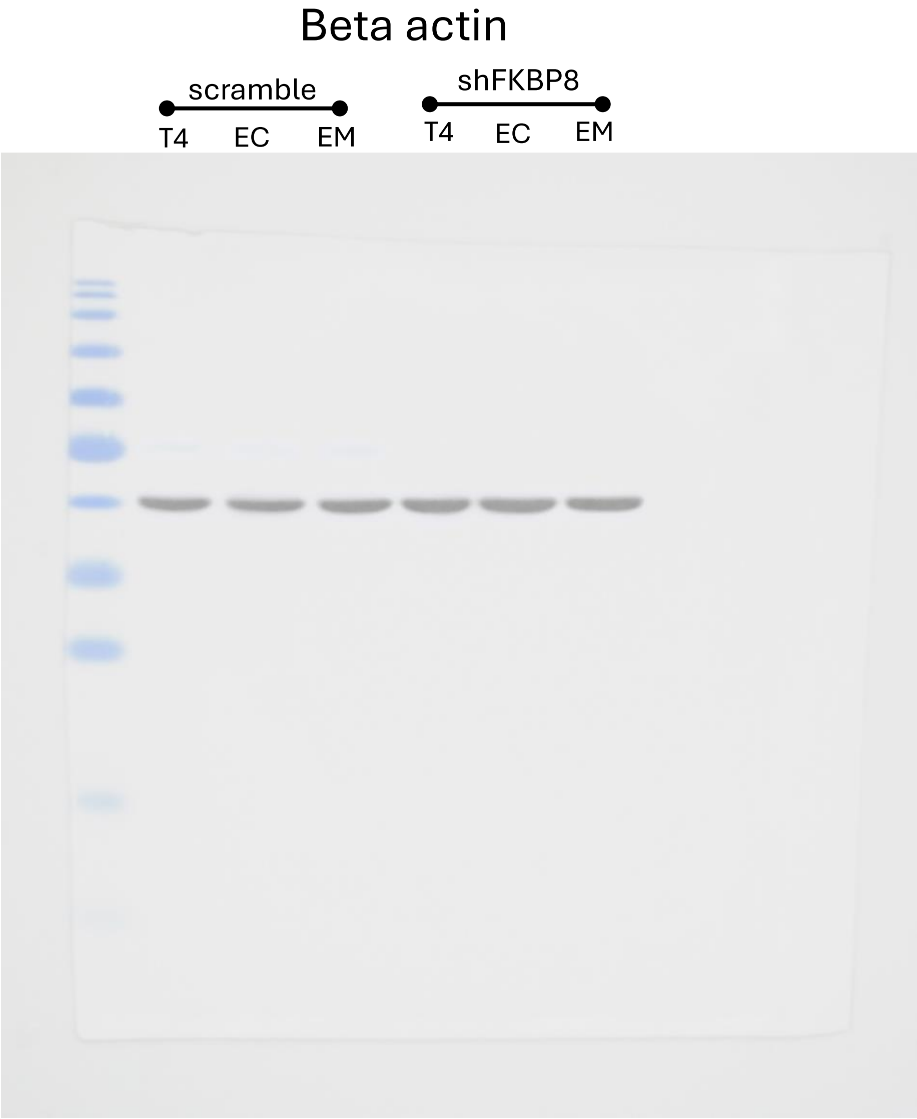

Uncropped supplemental blots

S2 Fig.

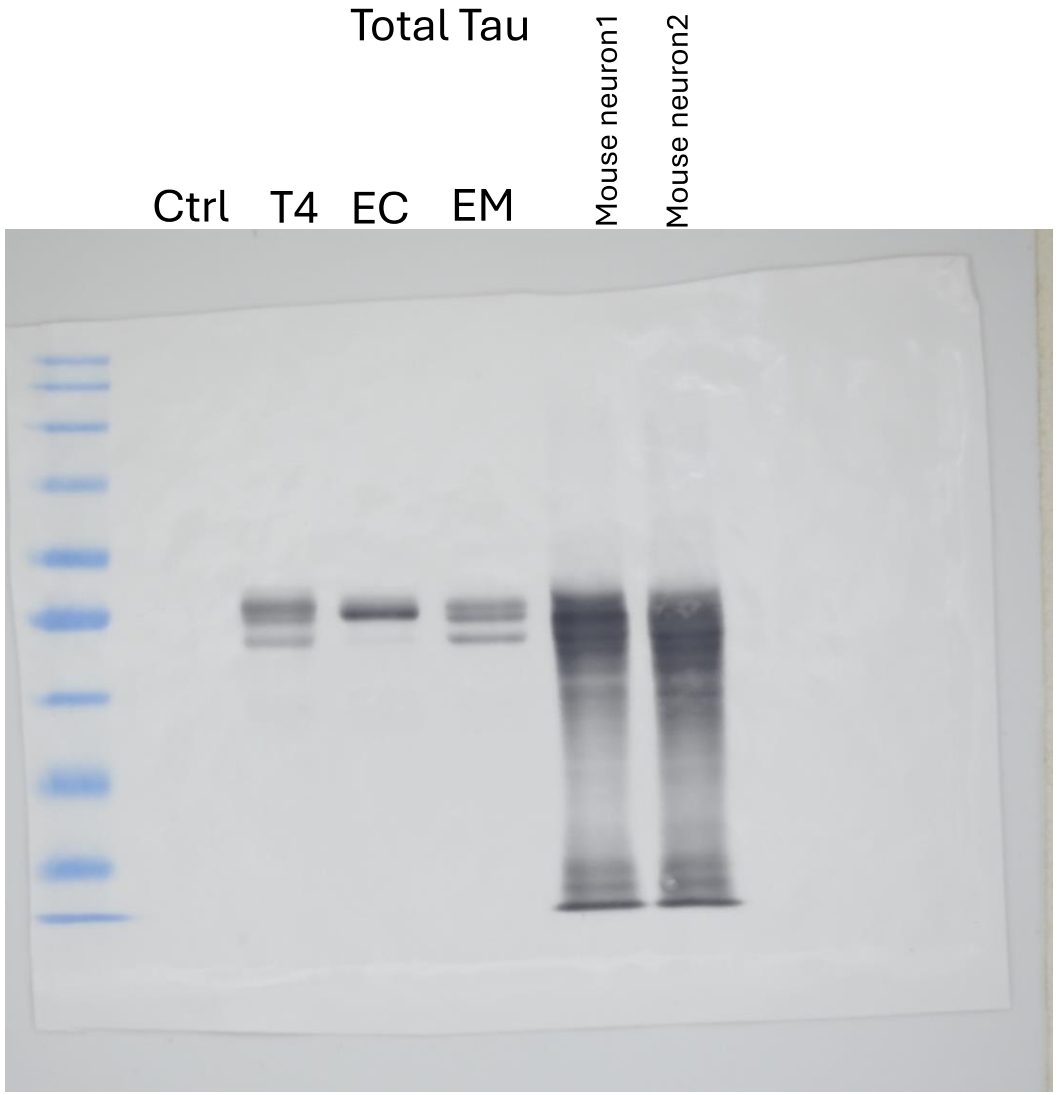
Total Tau
Ctrl
T4
EC
EM
Mouse neuron1
Mouse neuron2

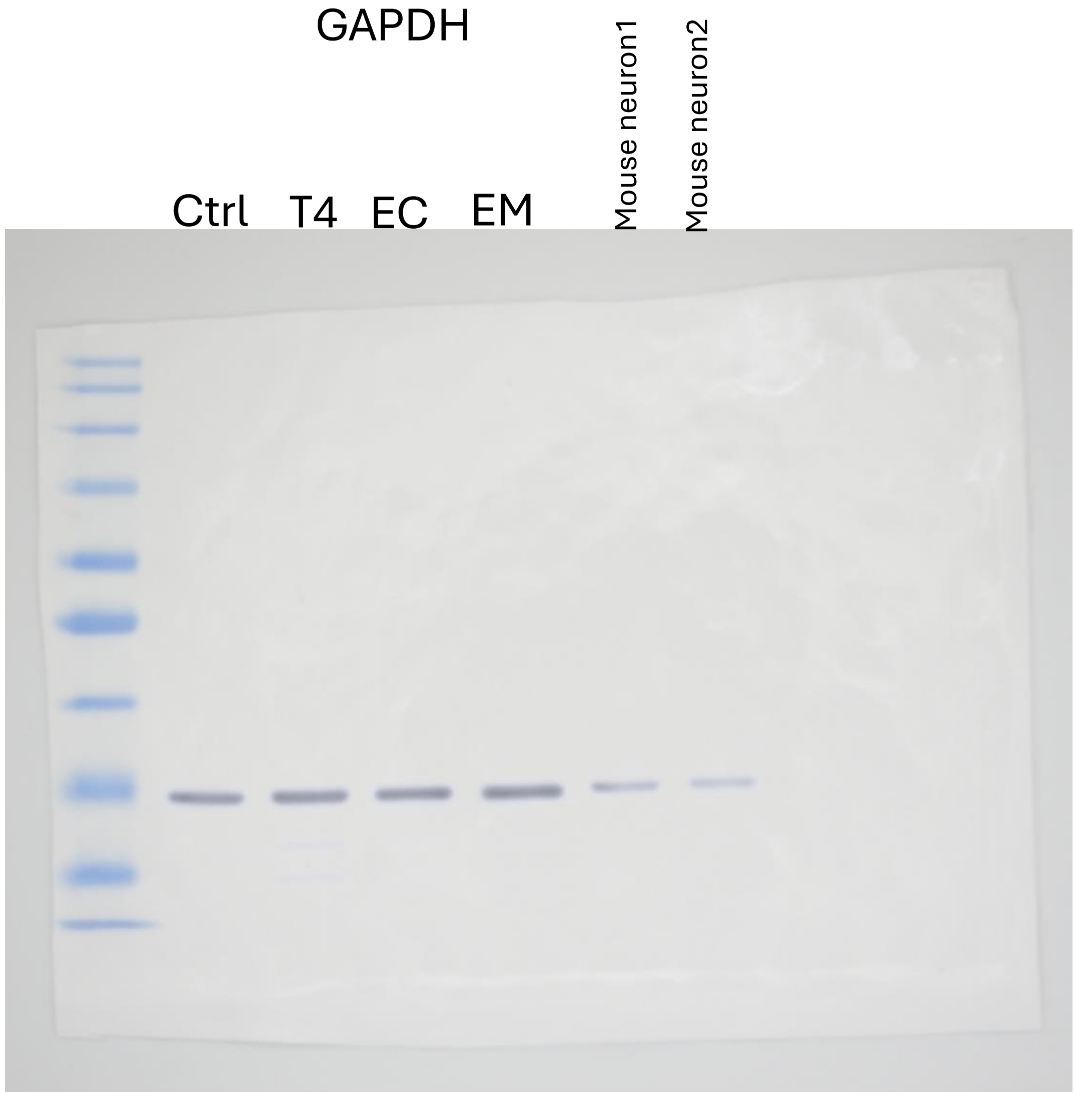
GAPDH
Ctrl
T4
EC
EM
Mouse neuron1
Mouse neuron2

S4B Fig.

FKBP8

ctrl — nocodazole — PQ — PQ nocodazole

T4 EC EM T4 EC EM T4 EC EM T4 EC EM

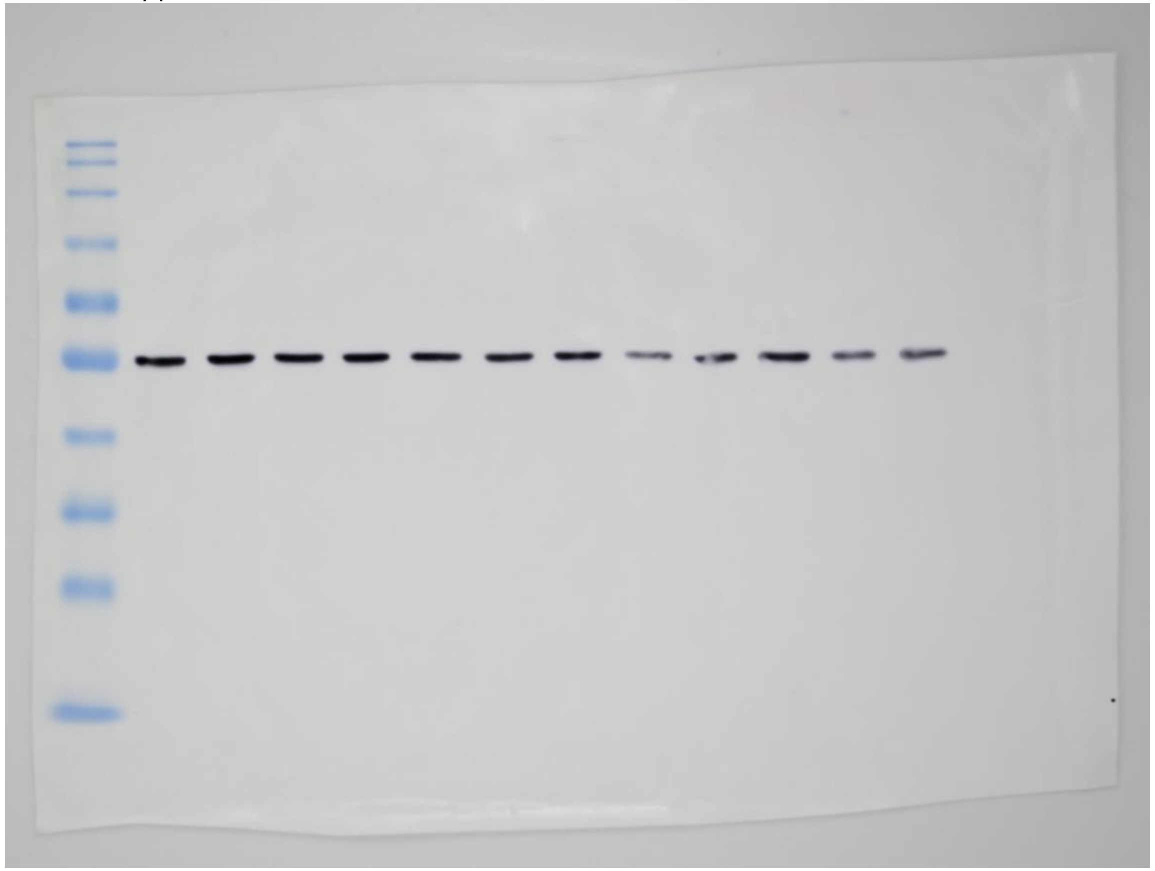

ctrl — nocodazole — PQ — PQ nocodazole

T4 EC EM T4 EC EM T4 EC EM T4 EC EM

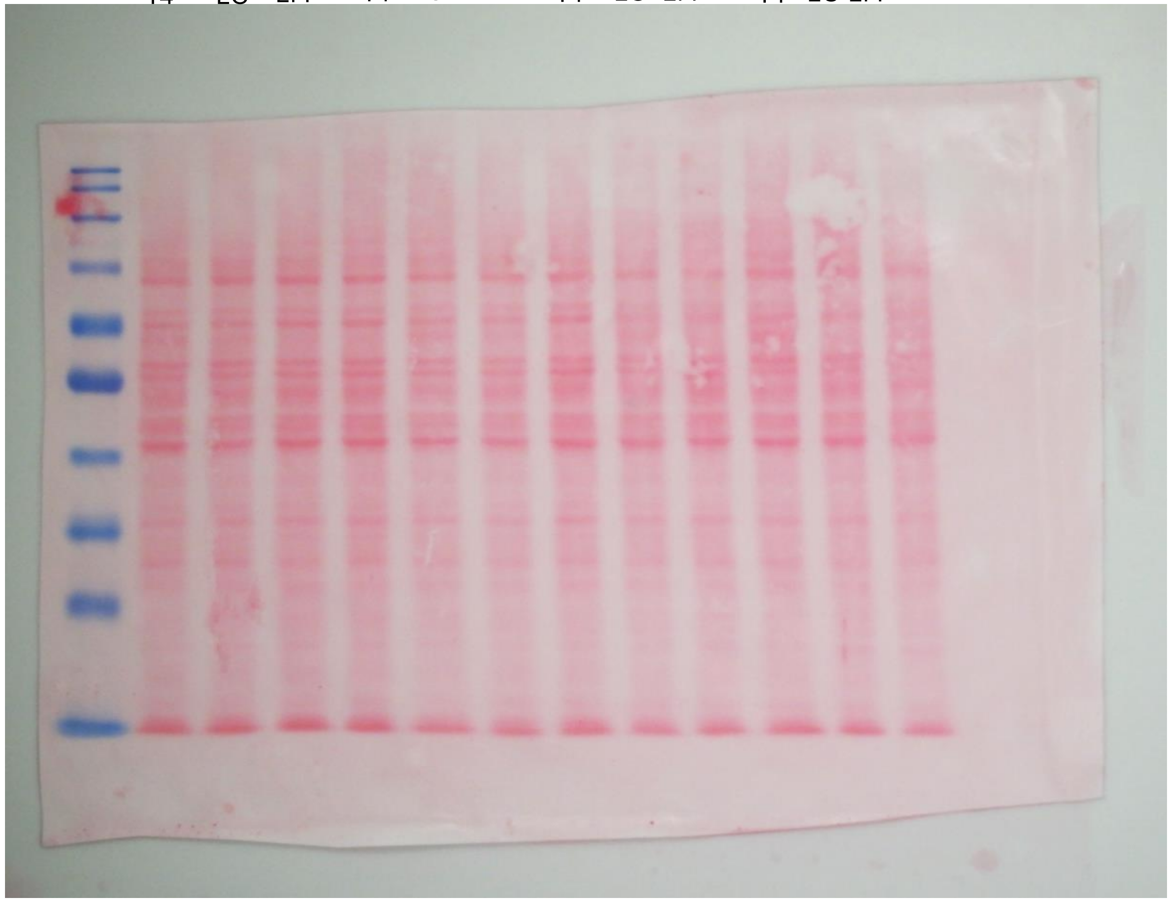

S6A and B Fig.

Total tau

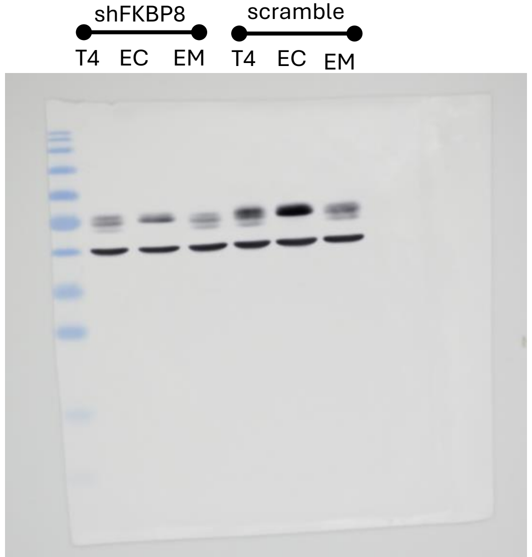

Experimental sample 1

Beta actin

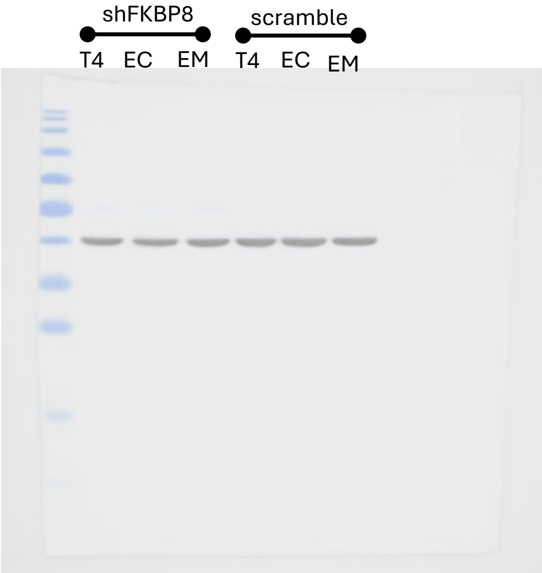

Experimental sample 1

Total tau

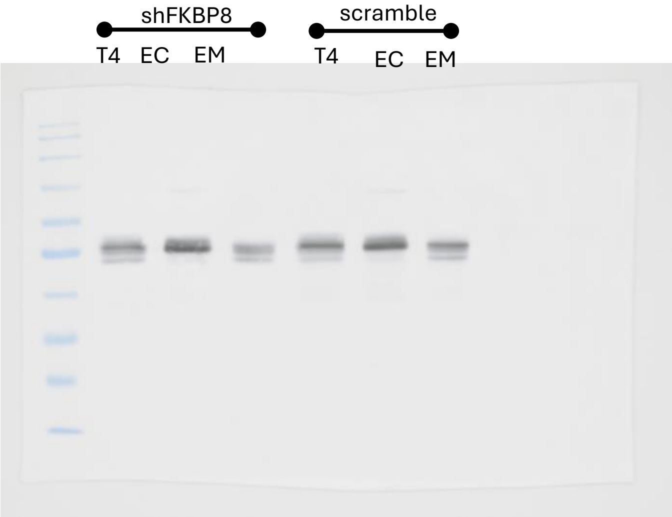

Experimental sample 2

Beta actin

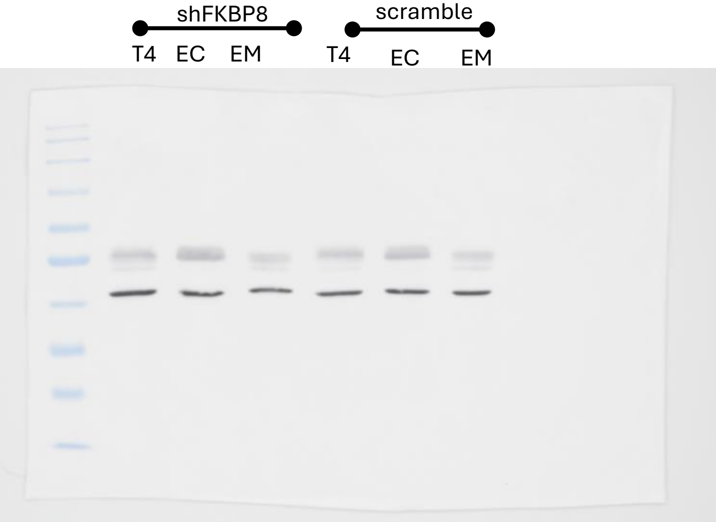

Experimental sample 2

S6A and B Fig.

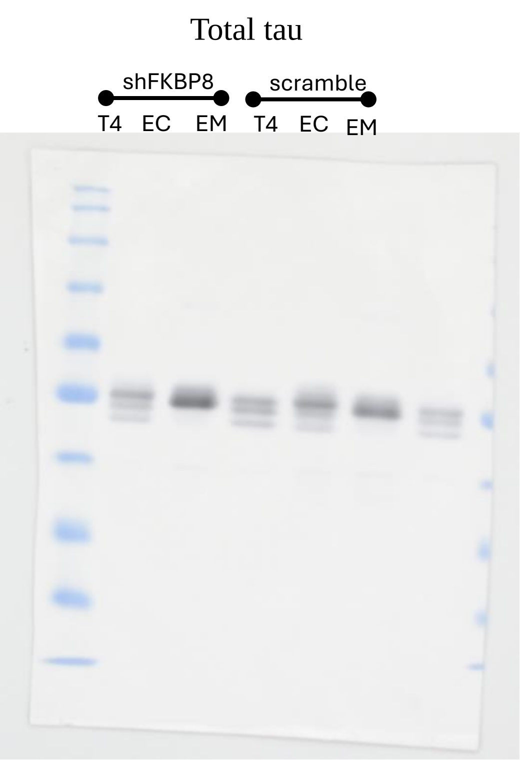

Experimental sample 3

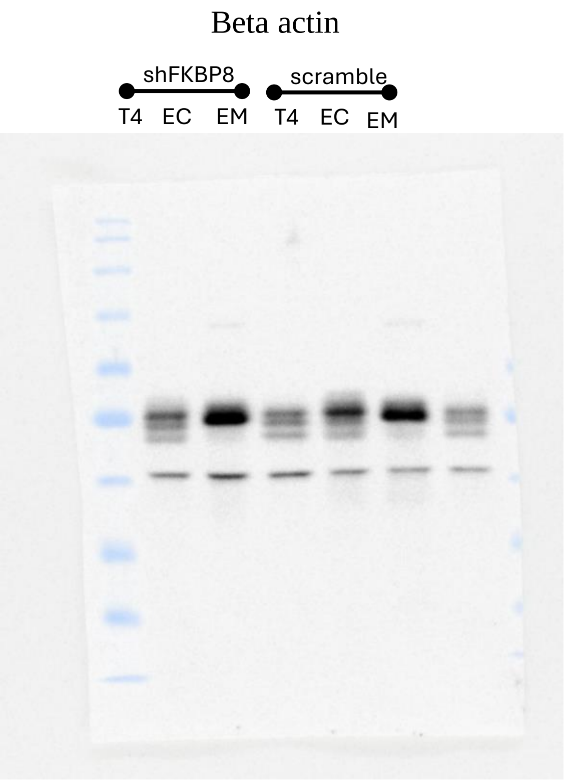

Experimental sample 3

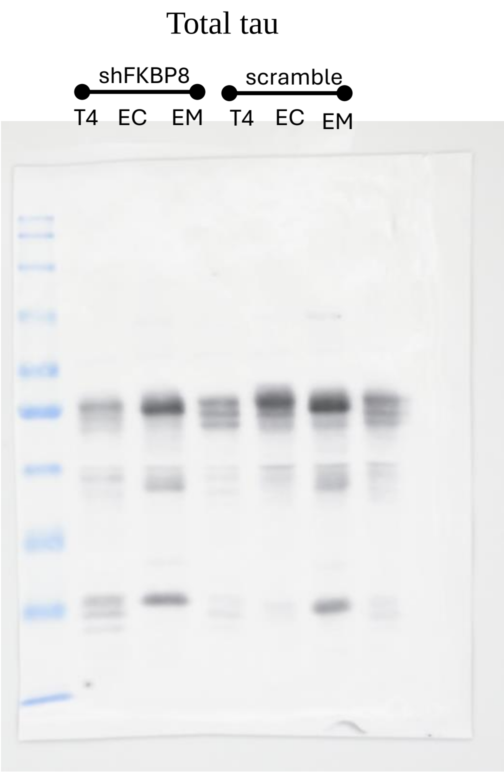

Experimental sample 4

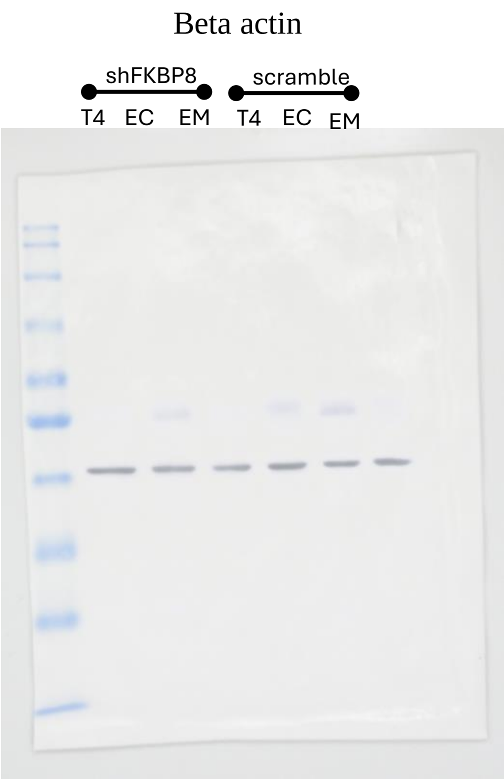

Experimental sample 4

S6A and B Fig.

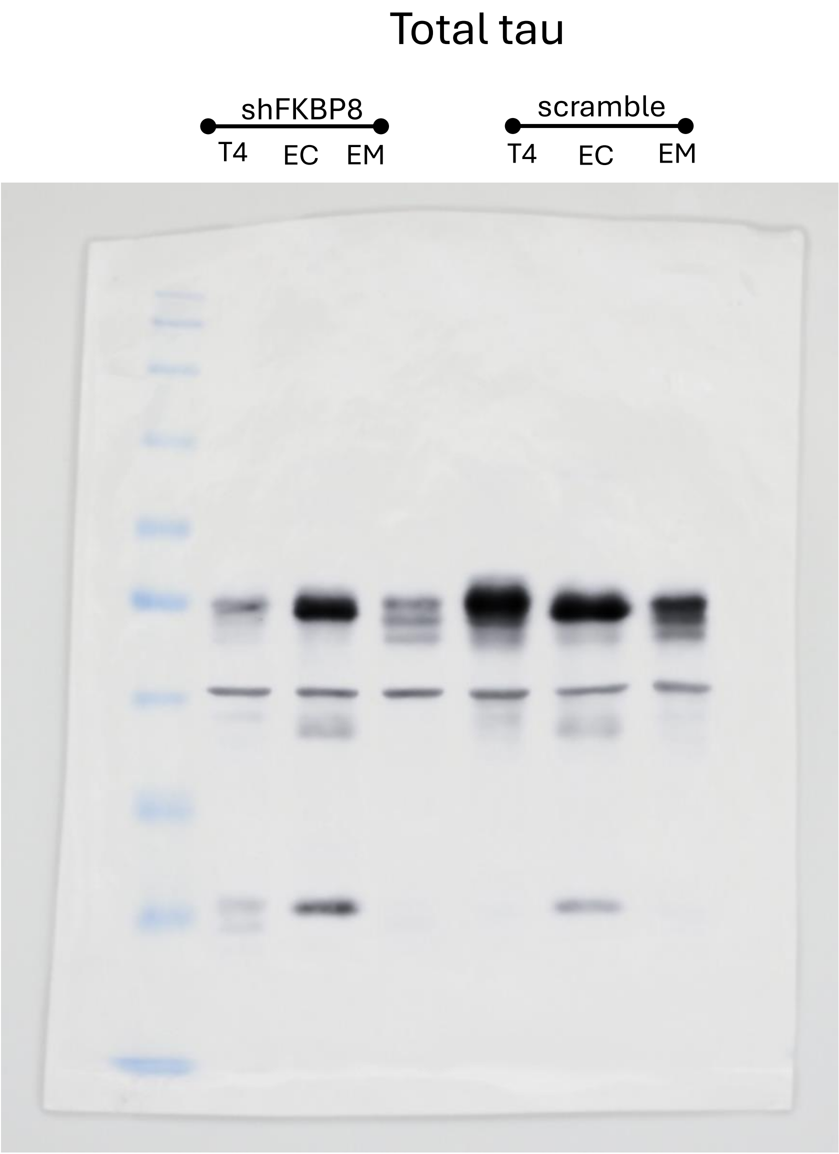
Total tau
shFKBP8
scramble
T4
EC
EM
T4
EC
EM

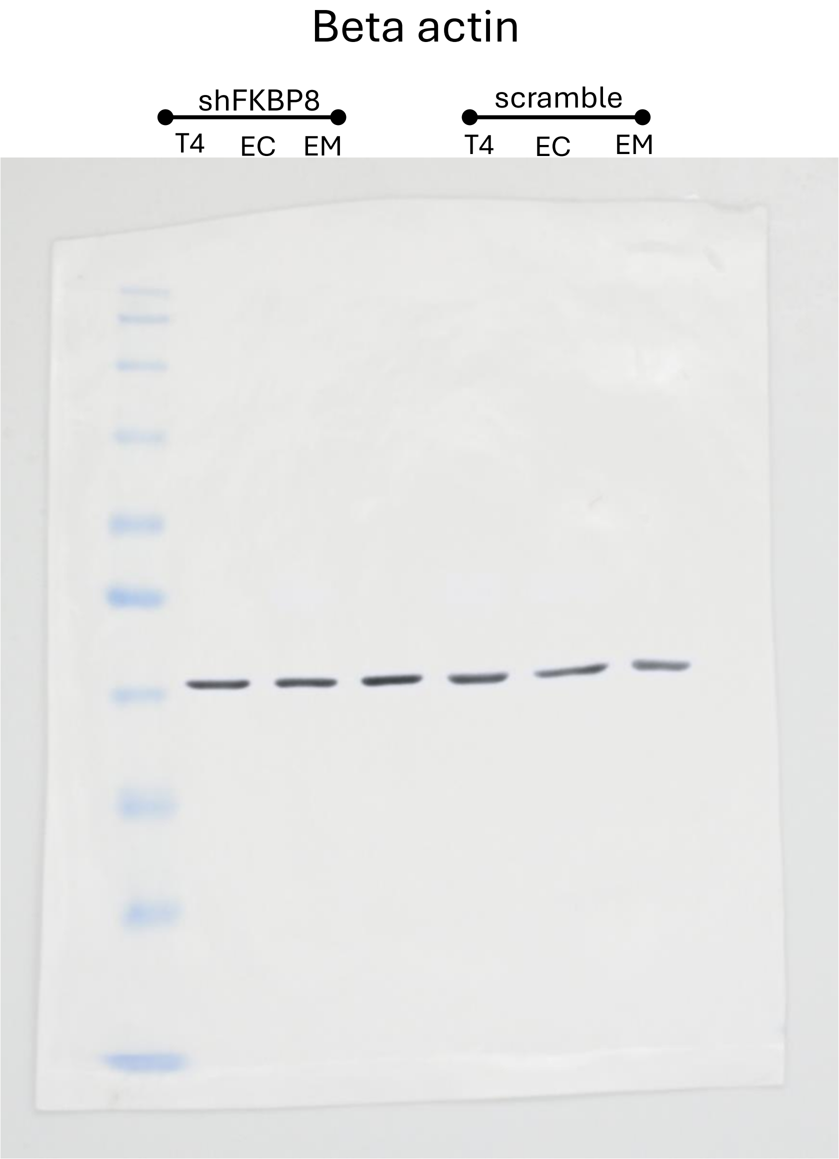
Beta actin
shFKBP8
scramble
T4
EC
EM
T4
EC
EM
